# Effectiveness of geriatric rehabilitation in inpatient and day hospital settings: a systematic review and meta-analysis

**DOI:** 10.1186/s12916-024-03764-7

**Published:** 2024-11-22

**Authors:** Eric K. C. Wong, Peter M. Hoang, Andrew Kouri, Sandeep Gill, Yu Qing Huang, Janice C. Lee, Sophie M. Weiss, Raymond Daniel, Jessie McGowan, Krystle Amog, Joanna E. M. Sale, Wanrudee Isaranuwatchai, David M. J. Naimark, Andrea C. Tricco, Sharon E. Straus

**Affiliations:** 1 Division of Geriatric Medicine, Department of Medicine, Temerty Faculty of Medicine, University of Toronto, Toronto, Canada; 2 Institute of Health Policy, Management and Evaluation, Dalla Lana School of Public Health, University of Toronto, Toronto, Canada; 3 Knowledge Translation Program, Li Ka Shing Knowledge Institute, St. Michael’s Hospital (Unity Health Toronto), 30 Bond Street, Toronto, ON M5B 1W8 Canada; 4 Division of Respirology, Department of Medicine, Women’s College Hospital, Toronto, ON Canada; 5 Institute of Health Policy, Management and Evaluation, Dalla Lana School of Public Health, University of Toronto, Toronto, Canada; 6 Temerty Faculty of Medicine, University of Toronto, Toronto, Canada; 7 School of Epidemiology and Public Health, Faculty of Medicine, University of Ottawa, Ottawa, Canada; 8 Musculoskeletal Health and Outcomes Research, Li Ka Shing Knowledge Institute, St. Michael’s Hospital, Unity Health Toronto, Toronto, Canada; 9 Department of Surgery, Faculty of Medicine, University of Toronto, Toronto, Canada; 10 Health Intervention and Technology Assessment Program (HITAP), Ministry of Public Health, Bangkok, Thailand; 11 Division of Nephrology, Department of Medicine, Sunnybrook Hospital, Toronto, Canada; 12 Keenan Research Centre of the Li Ka Shing Knowledge Institute, St. Michael’s Hospital, 30 Bond Street, Shuter 2-026, Toronto, ON M5B 1W8 Canada

**Keywords:** Geriatric rehabilitation, Inpatient, Day hospital, Older adults, Cognition, Mortality, Long-term care, Functional status

## Abstract

**Background:**

Geriatric rehabilitation is a multidisciplinary intervention that promotes functional recovery in older adults. Our objective was to assess the efficacy of geriatric rehabilitation in inpatient and geriatric day hospital settings.

**Methods:**

We searched MEDLINE, EMBASE, Cochrane Central Register of Controlled Trials, PsycINFO, PEDro and AgeLine from inception to September 30, 2022 for randomized controlled trials (RCTs) including older adults (age ≥ 65 years) undergoing geriatric rehabilitation (inpatient or day hospital) with a usual care comparator group. Primary outcome measures included mortality, long-term care home (LTCH) admission, and functional status. Secondary outcomes included discharge/remaining at home, functional improvement, length of stay, cognition, mood, and quality of life. Records were screened, abstracted and assessed for risk of bias (Cochrane Risk of Bias [RoB] 2) by two reviewers independently. We conducted a random effects meta-analysis to summarize risk ratios (RR, dichotomous outcomes) and standardized mean differences (SMD, continuous outcomes).

**Results:**

Of the 5304 records screened, 29 studies (7999 patients) met eligibility criteria. There were 23 RCTs of inpatient geriatric rehabilitation (6428 patients) and six of geriatric day hospital (1571 patients) reporting outcomes of mortality (26 studies), LTCH admission (22 studies), functional status (19 studies), length of stay (18 studies), cognition (5 studies), mood (5 studies) and quality of life (6 studies). The primary outcome of mortality at longest follow up was lower in the rehabilitation group (RR 0.84, 95% confidence interval [CI] 0.76 to 0.93, I^2^ = 0%). LTCH admission was lower in the rehabilitation group at longest follow up (RR 0.86, 95% CI 0.75 to 0.98, I^2^ = 8%). Functional status was better in the rehabilitation group at longest follow up (SMD 0.09, 95% CI 0.02 to 0.16, I^2^ = 24%). Cognition was improved in the rehabilitation group (mean difference of mini-mental status exam score 0.97, 95% CI 0.35 to 1.60, I^2^ = 0%). No difference was found for patient length of stay, mood, or quality of life.

**Conclusions:**

Geriatric rehabilitation in inpatient and day hospital settings reduced mortality, LTCH admission, and functional impairment. Future studies should explore implementation of this intervention for older adults.

**Review registration:**

PROSPERO: CRD42022345078.

**Supplementary Information:**

The online version contains supplementary material available at 10.1186/s12916-024-03764-7.

## Background

Rehabilitation aims to optimize function and decrease disability in patients with various health conditions [1]. The World Health Organization recognized the need for integrated, multidisciplinary, and universally accessible rehabilitation as part of its Rehabilitation 2030 strategy [2]. An aging population may contribute to increased disability globally [3], and thus, rehabilitation needs will continue to grow, particularly for older adults who are the primary users of rehabilitation [1]. Inpatient geriatric rehabilitation provides multidisciplinary care (including at least occupational therapy [OT] and/or physiotherapy [PT] [4]) to older adults with functional decline [5] such as those recovering from medical illness or surgery (e.g., hip fractures). Geriatric rehabilitation involves a tailored approach to progressively restore an older adult’s function, mobility and independence using various interventions (e.g., exercise, adaptive equipment, assistive devices, and modification of functional tasks) [6]. A systematic review of randomized controlled trials (RCTs) in 2010 showed that inpatient geriatric rehabilitation improved function (risk ratio [RR] 1.75, 95% confidence interval [CI] 1.31–2.35), reduced the need for long-term care home (LTCH) admission (RR 0.64, 95% CI 0.51–0.81), and reduced mortality (RR 0.72, 95% CI 0.55–0.95) [4] for older adults.

Geriatric rehabilitation resources are limited and patients who would potentially benefit from this intervention are often not provided with this treatment [7]. In a North American study of home care clients, 75.7% of older adults with rehabilitation potential did not receive OT or PT [8]. Policymakers have tried to replace more costly inpatient rehabilitation with outpatient geriatric rehabilitation where patients receive multidisciplinary care in an outpatient clinic or at home, but outcomes for patients are less positive, with a recent systematic review showing no difference in functional status, LTCH admissions or mortality [9]. The authors of the review of outpatient geriatric rehabilitation postulated that low participation (often not reported in outpatient trials) could be a reason for the lack of effect [9]. In light of the limited benefits of outpatient geriatric rehabilitation, providing an updated synthesis of the effectiveness of inpatient geriatric rehabilitation may help inform better allocation of resources.

Another mode of outpatient rehabilitation is via geriatric day hospitals, which provide intensive rehabilitation for older adults who attend on a regular basis for multidisciplinary care [10]. A systematic review of day hospitals was done in 2015 [11], which showed equivocal effectiveness because of (i) challenges in studying an appropriate population, (ii) a range of comparison interventions, and (iii) quality of the study designs [10, 11]. A comparison of day hospitals with a usual care control group was not done.

Through this systematic review and meta-analysis, we aim to update and summarize the effectiveness of geriatric rehabilitation in the inpatient and day hospital settings compared with usual care.

## Methods

This systematic review was registered with PROSPERO (CRD42022345078). The Cochrane Handbook for Systematic Reviews of Interventions was used to guide our methods [12] and the manuscript conforms to the Preferred Reporting Items for Systematic Reviews and Meta-Analyses (PRISMA) 2020 statement [13]. The study team consisted of scientists with experience in systematic reviews (ACT, SES), librarians (JM, RD), geriatricians (SES, EKCW, YQH, JCL), and scientists with expertise in clinical epidemiology (JEMS, WI, DN). Clinical experts (SES, EKCW) and methods experts (SES, ACT, JM, RD) were engaged from the protocol development stage through the end of the project. A patient partner was also engaged in selecting outcomes for the study.

### Eligibility criteria

We included RCTs of older adults (≥ 65 years [14]) that compared a geriatric rehabilitation intervention with usual care. We only included rehabilitation interventions designed specifically for older adults that included a multidisciplinary team with OT or PT care. We included rehabilitation interventions in an inpatient or day hospital setting. We excluded studies with rehabilitation in the community or at home. We also excluded studies with multidisciplinary geriatric care in an acute inpatient unit. Usual care was defined as the standard of care comparator that individual study authors used. We did not restrict by year of publication, language, or publication status.

### Information sources

We searched for RCTs indexed in Ovid MEDLINE (1946 to September 21, 2022), Ovid EMBASE (1974 to September 30, 2022), Ovid Cochrane Central Register of Controlled Trials (Issue 7, 2022), Ovid PsycINFO (1806 to September Week 1 2022), PEDro (2000 to September 21, 2022) and EBSCO AgeLine (1978 to September 21, 2022). The grey literature was searched to supplement the review using an approach that targeted key geriatric websites, journals, theses, and the CADTH Grey Matters checklist [15]. Conference proceedings were included. References of included articles and related systematic reviews were used to complete an exhaustive search.

### Search strategy

A comprehensive literature search was conducted by an experienced librarian (JM) first in the MEDLINE database and then translated to the other databases. The MEDLINE strategy was peer reviewed by a second librarian with expertise in systematic reviews using the Peer Review of Electronic Search Strategies (PRESS) checklist [16]. The search used a validated filter for RCTs (Cochrane) [17]. The search strategy is in Additional file 1: Appendix 1.

### Study selection process

We conducted screening by two independent reviewers working in pairs (PH, AK, SG, YQH, JCL, SMW, EKCW) for all records for level 1 (titles and abstract) and 2 (full text). A calibration exercise was done prior to level 1 screening using a sample of 50 titles and abstracts to ensure good agreement (> 80%). Discrepancies were mediated by a third reviewer (SES). We conducted a calibration exercise for level 2 screening using another sample of 50 full-text records from the eligible articles (> 80%). Study authors were contacted if study eligibility was unclear.

### Data collection process

Data were abstracted by two independent reviewers working in pairs (PH, AK, SG, EKCW) after a pilot abstraction exercise using a random sample of 3 studies (agreement of > 80%). Two reviewers independently abstracted all data using a data abstraction form. Discrepancies were resolved by discussion between the reviewers and a third reviewer (SES) was asked to review if needed.

### Data items

Abstracted data included study characteristics (e.g., location, author, year of publication, funding source), participant characteristics (e.g., mean age, sex, place of residence), intervention characteristics (e.g., setting, indication for rehabilitation, team members), and outcomes (e.g. mortality, LTCH admission, functional status). Population characteristics that promote equity were abstracted using the PROGRESS-Plus factors (e.g. place of residence, race, ethnicity, culture, language, occupation, gender, sex, religion, education, socioeconomic status, social capital) [18, 19]. Outcomes were recorded at discharge and at the longest available time for follow up data. The funding source of each study was recorded.

### Effect measures

The outcomes of interest were chosen after an informal discussion with a patient partner [20]. The primary outcomes included mortality, LTCH admission, and functional status (any measure). Secondary outcomes included returning/remaining home (number at home as defined by study author), functional improvement (as defined by study author), length of stay in rehabilitation, cognition (any measure), depression or anxiety (any measure), and quality of life (any measure). LTCH admission was defined as discharge to nursing homes, skilled nursing facilities, or care facilities. Functional status referred to an individual’s ability to do activities of daily living [4]. Primary outcomes were summarized on discharge and at longest follow up. The minimal clinically important difference (MCID) of continuous outcomes (functional status, cognition, mood, and quality of life) were reported in comparison with pooled estimates from the meta-analysis [21]. Pooled estimates were transformed back to natural units of the default measure (e.g. Barthel index for functional status) for comparison [22].

### Study risk of bias assessment

We assessed risk of bias of RCTs using the Cochrane Risk of Bias 2 tool [23]. Two reviewers working in pairs (PH, AK, SG, EKCW) independently assessed a sample of 5 trials, before independently reviewing the remaining studies once agreement was ≥ 80%.

### Synthesis methods

We descriptively summarized study characteristics, patient characteristics, risk of bias assessments, and frequencies of outcomes across included studies. We pooled primary and secondary outcomes in a meta-analysis using risk ratios (RR) to compare mortality, LTCH admission, discharge home, and functional improvement between geriatric rehabilitation and usual care [24]. Standardized mean differences (SMD) were used to compare functional, mood-related and quality of life changes. Mean differences (MD) were used to compare differences in length of stay and cognitive scores (all studies providing cognitive outcome data used the mini-mental status examination, MMSE [25]). Risk differences were calculated for the dichotomous outcomes to generate a number needed to treat (NNT) [26].

We assessed between-study statistical, clinical, and methodological heterogeneity. Clinical heterogeneity was assessed by looking at the population in individual studies, such as age, baseline cognitive status, and indication for rehabilitation. We explored methodological heterogeneity by examination of study design and risk of bias. If there was substantial statistical (I^2^ statistic > 60% [27]) or clinical/methodological heterogeneity and 10 or more included studies, we conducted a meta-regression analysis [12]. We employed subgroup analysis to explore the effects of clinical and methodological heterogeneity. Pre-specified subgroup analyses included attrition rate (< 10% versus ≥ 10%), indication for rehabilitation (hip fracture versus general geriatric rehab or other), age (mean age ≥ 80 years versus < 80), rehabilitation intervention (inpatient rehabilitation versus day hospital), cognitive status in eligibility criteria (dementia of any severity, mild to moderate dementia only, no dementia, or not reported), and length of follow up (< 6 months versus ≥ 6 months). Additional post-hoc subgroup analyses were done to explore team composition on outcomes with high heterogeneity as appropriate (OT, nurse, geriatrician, social worker and psychiatrist/psychologist). We conducted a sensitivity analysis by restricting to studies of low risk of bias only for the most at-risk domains for primary outcomes, which is a recommended approach to confirm robustness of the results [28]. We used random effects models [29] with 95% confidence intervals for the analysis. Between-study variance was estimated using the DerSimonian and Laird method [30]. Heterogeneity was quantified using the I^2^ statistic [31]. Meta-analysis was analyzed using the “meta” and “metafor” packages [32] in R [33].

### Reporting bias assessment

We assessed publication bias by visual inspection of contour-based funnel plots and by Egger’s regression [34].

### Certainty assessment

The certainty of evidence was assessed using the GRADE approach done by one investigator (EKCW) [35].

## Results

### Study selection

**Fig. 1:**
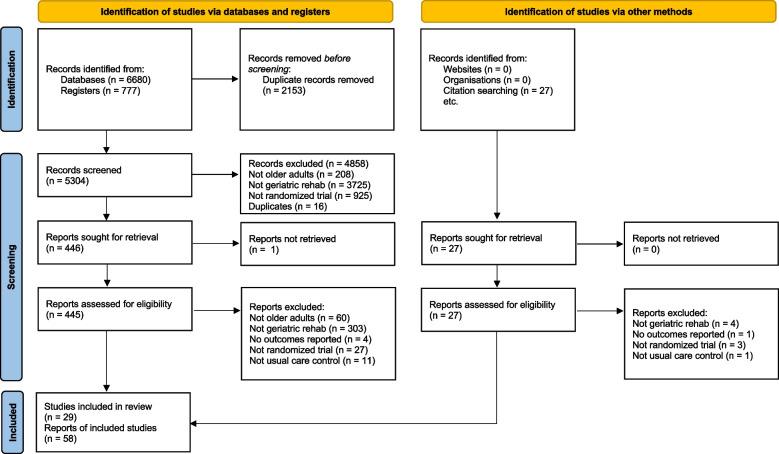
PRISMA 2020 flow diagram

We screened 5304 database records and reviewed 445 full-text reports for eligibility (Fig. 1). We included 27 primary studies from the screened records and two additional studies from searching references of included articles [36, 37] (total 29 studies, 7999 patients). One study was included in abstract form [38], and we were unable to contact the author for the full report.

### Study characteristics

We assessed inpatient geriatric rehabilitation in 23 studies (6428 patients) and geriatric day hospital in the remaining six studies (1571 patients). Eleven studies included general geriatric patients (medical or surgical mix), patients with hip fracture in 15 studies, patients with falls in one study, patients with heart failure in one study, and patients with stroke in one study (Table 1). The mean age across studies was 79.7 years (three studies did not report mean age, but were included based on age cutoff in eligibility criteria), and the mean proportion of females was 69.2% (1 study did not report sex). Gender was not reported in any study. The proportion of patients from home was 86.5% (23 studies reported) and from LTCH was 5.3% (21 studies reported). Some studies did not report all the categories of place of residence, so the proportions do not add up to 100%. Ethnicity or race were reported in four studies and education in seven studies (mainly high school or less). Income level, religion, occupation, socioeconomic status, and social capital were not reported in any studies.

**Table 1:** Characteristics of the included studies. Sorted by (i) inpatient geriatric rehabilitation and (ii) geriatric rehabilitation at day hospital

Author and year	Patient	Intervention	Comparator	Setting	Team members	Sample size (I/C)	Risk of bias
**Inpatient geriatric rehabilitation**							
Applegate 1990	Medical/surgical mix, age > 65	Geriatric assessment unit, a 10-bed unit in a rehabilitation hospital	Usual care (47% home health care and 22% rehab units)	Rehabilitation hospital, USA	PT, OT, RN, SW, MD, SLP, audiology, dietitian, psychologist	78/77	High risk
Cohen 2002	Medical/surgical mix with frailty, age > 65, excluded LTC	2 × 2 factorial. Inpatient geriatric unit x outpatient geriatric clinic. Twice weekly team meetings	Usual care inpatient x usual care outpatient	11 VA medical centres, USA	RN, SW, geriatrician. PT, OT, dietitian, pharmacist on request	694/694	Low risk
Fleming 2004	Medical/surgical mix, age > 65	Rehabilitation service based in social services old people’s homes	Usual health and social care	Residential facilities with rehab services in Nottingham, UK	OT/rehab assistants for all. PT, RN, MD on request	81/84	Low risk
Hinkka 2007	Age > 65, community dwelling, functional decline, at risk of LTC in next 2 years	Multidisciplinary geriatric rehab program. Three inpatient periods (5, 11, and 5 days) over a period of 8 months. Includes CGA, rehab, classes, recreation, physical activation	Usual local social and health care services	Network of rehabilitation hospitals, local health service providers, and community organization/ churches, Finland	MD, PT, SW, OT	321/324	Some concerns
Kakutani 2018	Age ≥ 65 with admission for acute heart failure	Critical pathway, including ADL rehab, physical exercise, education (medication adherence, smoking, sodium, weight etc.). Includes multidisciplinary component. Detailed in separate paper (Circ Rep 2019; 1: 123–130)	Usual care, multidisciplinary care team, including PT	Japan	PT, dietitian, pharmacist, RN, MD	50/64	Some concerns
Karppi 1995	Medical/surgical mix, age > 65, community dwelling in supervised home care population	Comprehensive multidisciplinary geriatric assessment in an inpatient geriatric assessment unit	Usual home care	Joint ward (8 beds) with a rehabilitation unit (8 beds), Finland	PT, OT, RN, SW, PSW, geriatrician, psychiatrist	104/208	High risk
Rubenstein 1984	Medical/surgical mix, age > 65	Geriatric evaluation unit (15 beds on a 29-bed ward in intermediate-care area of the hospital, not acute care)	Usual care through the acute care process, no outpatient geriatric follow up	Veterans Centre, USA	Geriatrician, PT, OT, RN, SW, PSW, audiology, geriatric dentist, psychologist, public health nurse, physician assistant	63/60	Some concerns
Saltvedt 2002	Acute medical, age > 75	Geriatric evaluation and management unit. Comprehensive assessment, prevention of complications by interdisciplinary approach. Early mobilization, optimize ADLs, early rehabilitation	Usual care	University hospital, Norway	PT, OT, RN, geriatrician, dentist. SW on request	127/127	Some concerns
Cameron 1993	Hip fracture, age > 50 (Meta-analysis only included those not from LTC)	“Accelerated rehab” LTC patients: quick discharge and visiting PT for LTC rehab Others: 2xPT per day, 5 days per week plus multidisciplinary care	Usual care (LTC patients discharged when orthopedically appropriate; other patients referred to rehab and geriatrics a few days after surgery; then rehab ward)	Hospital ward, Australia	For LTC patients: only PT For others: PT, OT, RN, SW on request, geriatrician, physiatrist, dietitian on request	79/79	Some concerns
Fordham 1986	Hip fracture, female only, age > 65	Orthogeriatric joint management in an acute care unit and a postacute rehab unit, if a longer stay was needed. Team rounds once a week and joint decision making regarding transfer and discharge	Usual orthopedic care	St. Luke's Hospital (long stay hospital) and District General Hospital in York, UK	Geriatrician and orthopedic doctors, RN, PT, OT, SW	50/58	High risk
Gilchrist 1988	Hip fracture, female only, age > 65	Orthogeriatric unit with weekly team rounds	Separate ward with no case conference or routine geriatric consult	General hospital in Glasgow, UK	Weekly rounds by geriatrician, orthopedic resident, RN, PT, OT, SW	97/125	Some concerns
Huusko 2000	Hip fracture, age > 64	Geriatric ward for about 2 weeks of intensive rehabilitation to promote early ambulation, self-motivation and function; PT twice a day	“Local hospital wards for standard care”	Geriatric ward in general hospital, Finland	PT, OT, RN, SW, geriatrician, physiatrist, neurologist, psychiatrist	120/123	High risk
Kennie 1988	Hip fracture, female only, age > 65	Geriatrician ward rounds twice a week plus a weekly multidisciplinary case conference. Same PT/OT, orthotic services as control group	Stayed on ortho ward or short stay ward. Got PT, OT, orthotic services etc	Separate hospital orthopedic ward 5km away in Stirling, UK	Geriatrician, GP, PT, OT, orthotic	54/54	Low risk
Naglie 2002	Hip fracture, age > 70 from community and LTC	Multidisciplinary card ward for older adults staffed by geriatrician with early mobilization. Separate ward in acute care hospital PT twice a day, Monday to Friday	Separate usual care ward. Allied health and geriatric consult upon request	University general hospital, Canada	Geriatrician, RN, PT, OT, CNS, SW	141/138	Low risk
Prestmo 2015	Hip fracture, age > 70 from community only (excluded LTC)	Multidisciplinary comprehensive geriatric ward, including geriatric syndromes, mental health, functional status, social situation, early discharge planning and early mobilization and initiation of rehab	Orthopedic card in orthopedic trauma ward, staffed by ortho surgeon, RN, PT (No OT in control group)	Central hospital, Norway	Geriatrician, RN, PT, OT	198/199	Low risk
Sanchez Ferrin 1999	Hip fracture, age > 64	Geriatric functional unit, geriatric assessment and team meeting, could directly consult rehabilitation service for admission even if trauma service did not request it	Usual care from trauma service with consultations on request. Trauma service initiates rehab request	Hospital in Barcelona, Spain	Geriatrician, nurse, SW, PT, psychologist	103/103	Some concerns
Shyu 2005	Hip fracture, age > 60	Geriatric consultation, rehabilitation program and discharge-planning service (3 part intervention). Includes in home rehab after hospitalization	Usual care, variable PT coverage depending on insurance. Discharge home after 7 days, no in-home rehab	Large hospital, Taiwan	PT, RN, geriatrician, physiatrist	80/82	Some concerns
Shyu 2013	Hip fracture, age ≥ 60, excluded LTC	**3-arm study**, with 2 intervention groups 1. Multidisciplinary care, including geriatric consultation, rehabilitation program (daily PT), discharge planning with post-hospital services 2. Comprehensive care model, above components PLUS in-home rehab, nutritional consultation, depression management, falls prevention	Usual care, including nursing and PT care with discharge around 5–7 days	Large hospital, Taiwan	Geriatrician, PT, RN, dietitian, psychiatrist	200/99	Some concerns
Stenvall 2007	Hip fracture, age > 70	Geriatric ward with a special intervention program, early mobilization, treatment of geriatric syndromes, multidisciplinary care	Conventional care in an orthopedic ward. Can be transferred to separate geriatric unit for longer rehab	Hospital, Sweden	PT, OT, RN, geriatrician	102/97	Some concerns
Swanson 1998	Hip fracture, age > 55	Early surgery, minimal narcotic analgesia, intense daily therapy and close monitoring of patient needs via a multidisciplinary approach. Twice a day PT; daily OT/SW	Standard orthopedic management. Geriatrician consult on request. Daily PT. OT/SW on request	Orthopedic ward of teaching hospital, Australia	PT, OT, SW, geriatrician	36/31	Some concerns
Tseng 2019	Hip fracture, age ≥ 60	Multidisciplinary geriatric rehab (geriatrics consultation, in hospital and in home rehab) plus diabetes care (diet, BP, dyslipidemia, education). Daily PT	Usual care, including nursing and PT care with discharge around 5–7 days	Large hospital, Taiwan	Geriatrician, PT, RN, dietitian	88/88	Some concerns
Vidan 2005	Hip fracture, age > 65	Geriatrician MRP daily round, rehab specialist coordinated PT. Weekly case conference	Usual care on same orthopedic wards as intervention group. Managed by surgeon and nurses	Orthopedic ward in a university hospital, Spain	Geriatrician, PT, RN, SW, rehab doctor	155/164	Some concerns
Watne 2014	Hip fracture, no age cutoff	Orthogeriatric ward, including CGA, daily team meetings, early intensive mobilization, nutrition, discharge planning	Usual care on orthopedic ward	Oslo University Hospital, Norway	Geriatrician, nurse, PT, OT, nursing assistant. Dietitian and SW on request	163/166	Some concerns
**Geriatric day hospital rehabilitation**							
Conroy (Masud) 2010	Age > 70 with falls, from GP practices	Falls prevention program in geriatric day hospital	Usual care (can be referred to other falls prevention exercise programs, all outpatient)	8 rural and urban GP practices, East Midlands, UK	PT, OT, geriatrician	183/181	Low risk
Eagle 1991	Age > 65, either referred from community or prior to discharge from acute care hospitalization	Day hospital, 2 days/week, each visit 4–5 h. Multidisciplinary care and rehab	Usual care (inpatient GAU/outpatient clinic/early discharge and community follow up)	Chedoke-McMaster Day Hospital in Canada	PT, RN, SW, SLP, MD with training in geriatrics. Dietitian and pharmacist on request	55/58	Some concerns
Hui 1995	Acute stroke age > 65. Excluded dementia	Geriatrician MRP in hospital and geriatric day hospital after discharge	Neurologist MRP in hospital and usual outpatient clinic after discharge	New Territories East region, Hong Kong	Multidisciplinary but no details	59/61	Some concerns
Tucker 1984	Age > 55 requiring rehab	Day hospital, attended 2–3 days per week, 5.5h/visit, for 6–8 weeks	Usual care (inpatient, outpatient, domiciliary, GP, day centre, as before day hospital program)	New Zealand	PT, OT, RN, SW, MD, SLP	62/58	Some concerns
Weissert 1980	Medicare eligible patients with functional impairment without 24h care need	Day care with strong health care orientation and physical rehabilitation	Usual care	4 USA cities: 2 in New York, 1 in Kentucky and 1 in California	PT, OT, RN, SW, SLP, audiology, dietitian, eye care, podiatry	253/270	High risk
Woodford 1962	Postacute older adults, age > 60, living alone in community, previous inpatients at a Geriatric Unit. With physical limitations	Day hospital, 1 day/week, 8 h per visit	Usual care	Sunderland, UK	PT, OT, MD, volunteers, admin staff	168/163	High risk

*LTC* long-term care, *CGA* comprehensive geriatric assessment, *PT* physiotherapist, *OT* occupational therapist, *RN* registered nurse, *SW* social worker, *MD* medical doctor, *SLP* speech language pathologist, *CNS* clinical nurse specialist, *I/C* number of participants in the intervention and control groups

Nearly all studies included a PT (*n* = 28, 96.6%) as part of the intervention. An OT was part of the team in 21 studies (72.4%), a nurse in 23 studies (79.3%), a geriatrician in 20 studies (69.0%), and a social worker in 18 studies (62.1%). The models of care were diverse and frequency of PT or OT visits were not consistently reported. A summary of all outcomes is shown in Table 2.

**Table 2:** Summary of outcomes. The summary estimates of all outcomes reported in this meta-analysis. Asterisk (*) denotes statistical significance

**Outcome**	**No. of patients (studies)**	**RR/SMD/MD (95% CI)**	**I**^**2**^** (%)**	**Certainty of evidence (GRADE)**
Mortality at longest follow up	7619 (26 studies)	RR 0.84 (0.76 to 0.93)*	0	⊕⊕⊕⊝ Moderate due to risk of bias and imprecision◇‡
Mortality at discharge	2968 (15 studies)	RR 0.69 (0.49 to 0.95)*	0	⊕⊕⊕⊝ Moderate due to risk of bias◇
LTC admission at longest follow up	6891 (22 studies)	RR 0.86 (0.75 to 0.98)*	8	⊕⊕⊕⊝ Moderate due to risk of bias and imprecision◇‡
LTC admission at discharge	2600 (13 studies)	RR 0.80 (0.65 to 0.97)*	0	⊕⊕⊕⊝ Moderate due to risk of bias◇
Functional status at longest follow up	6052 (19 studies)	SMD 0.09 (0.02 to 0.16)*	24	⊕⊕⊕⊝ Moderate due to risk of bias, imprecision and indirectness◇§¶
Functional status at discharge	2364 (7 studies)	SMD 0.28 (0.05 to 0.50)*	67	⊕⊕⊝⊝ Low due to risk of bias, imprecision and indirectness◇§¶†
Functional improvement at longest follow up	2390 (11 studies)	RR 1.37 (1.20 to 1.56)*	20	⊕⊕⊕⊝ Moderate due to risk of bias◇
Functional improvement at discharge	1087 (5 studies)	RR 1.56 (1.16 to 2.09)*	0	⊕⊕⊕⊕ High
Discharge home	2077 (11 studies)	RR 1.26 (1.03 to 1.54)*	63	⊕⊕⊝⊝ Low due to risk of bias and inconsistency◇†
Remain home at longest follow up	1991 (10 studies)	RR 1.15 (1.01 to 1.32)*	53	⊕⊕⊝⊝ Low due to risk of bias, imprecision and inconsistency◇‡†
Length of stay	5028 (18 studies)	MD 0.79 days (-4.74 to 3.17)	91	⊕⊕⊝⊝ Low due to risk of bias, imprecision and inconsistency◇‡†
Cognition at longest follow up	1824 (5 studies)	MD 0.97 points (0.35 to 1.60)*	0	⊕⊕⊕⊝ Moderate due to imprecision§
Mood at longest follow up	1209 (5 studies)	SMD -0.67 (-2.30 to 1.05)	99	⊕⊕⊝⊝ Low due to risk of bias, imprecision, indirectness, and inconsistency◇‡¶†
Quality of life at longest follow up	3004 (6 studies)	SMD 0.09 (-0.11 to 0.28)	56	⊕⊕⊝⊝ Low due to imprecision, indirectness, and inconsistency‡¶†

*RR* risk ratio, *95% CI* 95% confidence interval, *I*^*2*^ measure of heterogeneity, *SMD* standardized mean difference, *MD* mean difference

◇Some older studies did not indicate blinded outcome assessments or had missing outcome data

‡Confidence interval includes both possibility of harms and benefits in the inpatient and/or day hospital settings

§Difference in outcome does not meet minimal clinically important difference (MCID)

¶Use of different scales for measurement

†High statistical heterogeneity

### Risk of bias in studies and publication bias

**Fig. 2:**
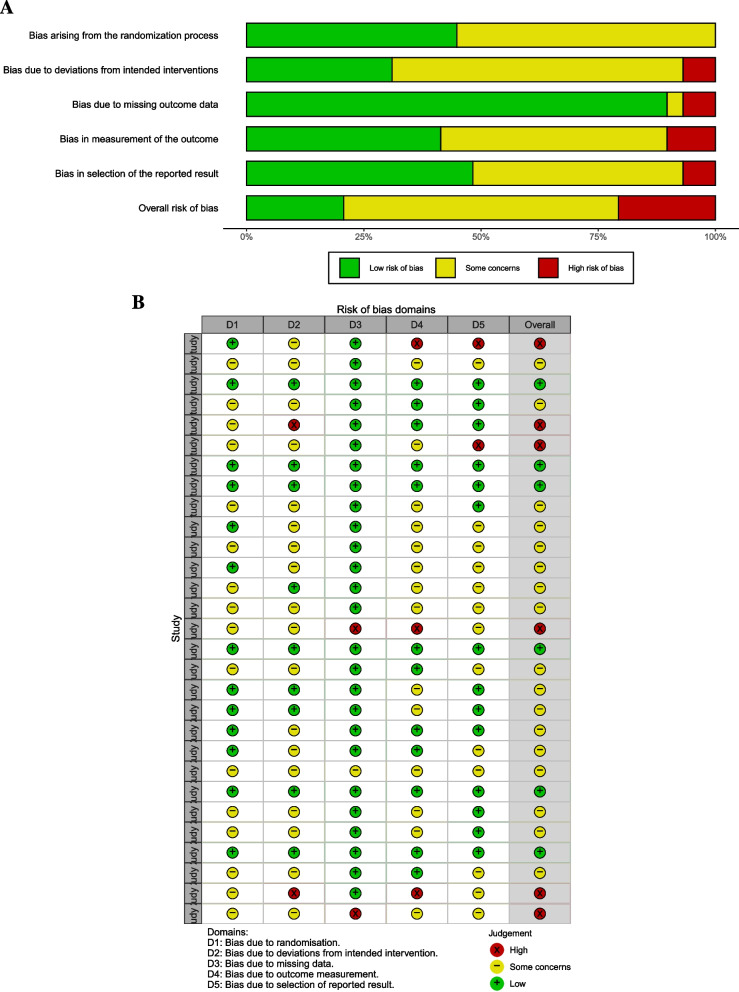
**a** Weighted bar plots of risk-of-bias judgements within each bias domain and (**b**) traffic light plot of risk of bias domain judgements for each included study

**Fig. 3:**
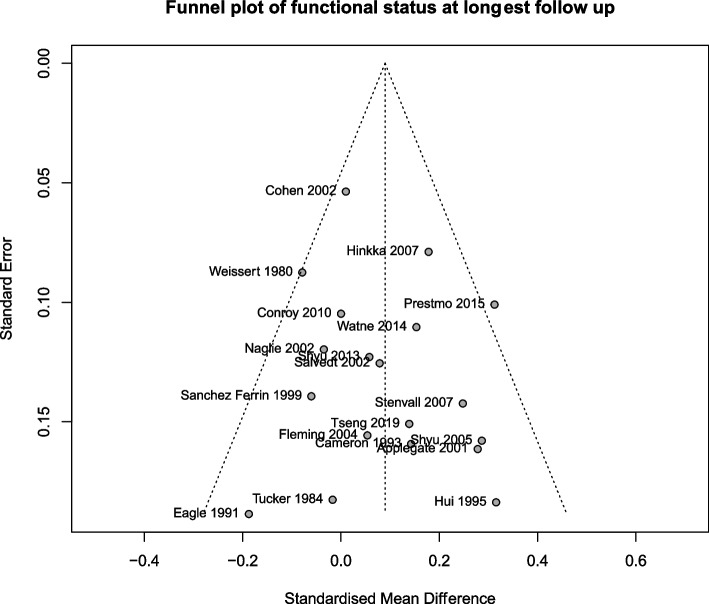
Funnel plot of functional status at longest follow up

In the risk of bias assessment, six studies were high risk, 17 had some concerns, and six were low risk (Fig. 2). A funnel plot of the functional status outcome at longest follow up is shown in Fig. 3, which did not demonstrate asymmetry. Egger’s test did not reveal publication bias (intercept 0.76 [95% CI −0.69 to 2.22], *p* = 0.32).

## Results of syntheses

### Primary outcomes: mortality, LTCH admission and functional status

**Fig. 4:**
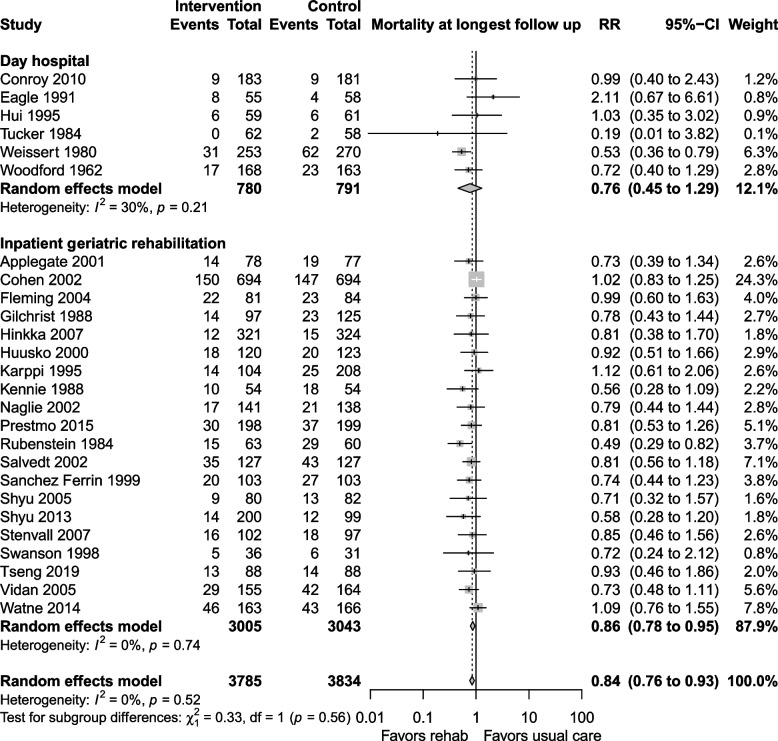
Mortality at longest follow up

The certainty of the evidence for mortality was moderate, downgraded due to risk of bias and imprecision. Mortality at longest follow up (Fig. 4) was reported in 26 studies, with lower mortality in the geriatric rehabilitation group, RR 0.84 (95% CI 0.76 to 0.93, I^2^ = 0%) compared with usual care. Both inpatient geriatric rehabilitation (RR 0.86, 0.78 to 0.95, I^2^ = 0%) and day hospitals (RR 0.76, 0.45 to 1.29, I^2^ = 30%) reduced mortality at longest follow up, but the day hospital subgroup had confidence intervals that could not exclude an increase in mortality. In absolute terms, the risk difference for mortality at longest follow up was −0.03 (−0.04 to −0.01), which translated to a NNT of 33 (25 to 100).

Subgroup analysis by indication for rehabilitation revealed that general geriatric rehabilitation and rehabilitation for participants with hip fracture benefitted similarly but only those with hip fractures had a significant reduction in mortality (RR 0.82, 0.73 to 0.92, I^2^ = 0%). Studies with a population with mean age ≥ 80 years had a significant mortality reduction (RR 0.84, 0.75 to 0.94, I^2^ = 0%) compared with studies with population with mean age < 80 years. Subgroup analysis of studies reporting outcomes at 6 months or less (RR 0.77, 0.64 to 0.92, I^2^ = 0%) and follow up over 6 months (RR 0.83, 0.73 to 0.94, I^2^ = 15%) was similar. Forest plots for sensitivity analyses, subgroup analyses, and mortality at discharge are shown in Additional file 1: Appendix 2–3.

**Fig. 5:**
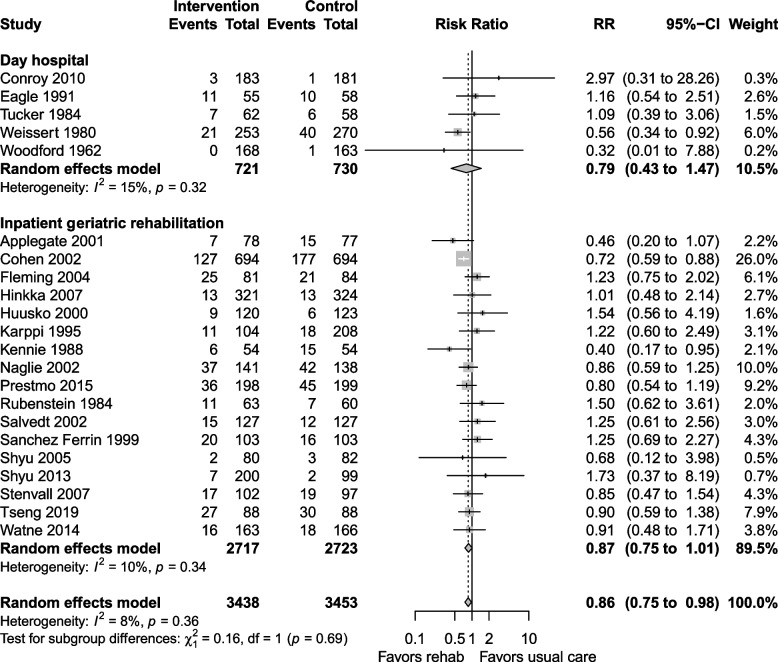
LTCH admission at longest follow up

The certainty of the evidence for LTCH admission was moderate, downgraded for risk of bias and imprecision. LTCH admission at longest follow up was reported in 22 studies, with a RR 0.86 (0.75 to 0.98, I^2^ = 8%) favouring geriatric rehabilitation (Fig. 5). Day hospital and inpatient rehabilitation settings reduced LTCH admissions, but the subgroup estimates had wide confidence intervals that could not exclude an opposite effect (Fig. 5). The risk difference was −0.01 (−0.03 to 0.01) and the NNT was 100 (−100 to 33).

**Fig. 6:**
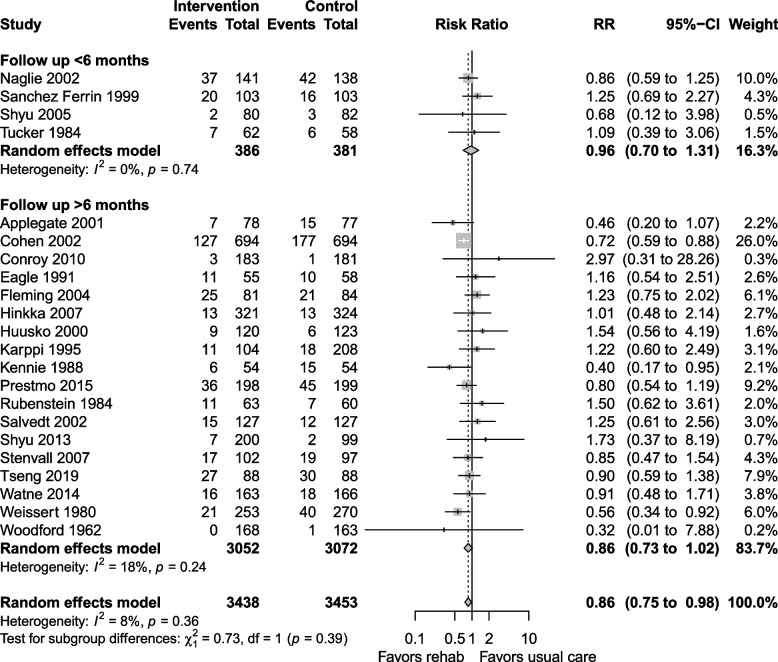
Subgroup analysis for LTCH admission at longest follow up: follow up ≤ 6 months vs. > 6 months

**Fig. 7:**
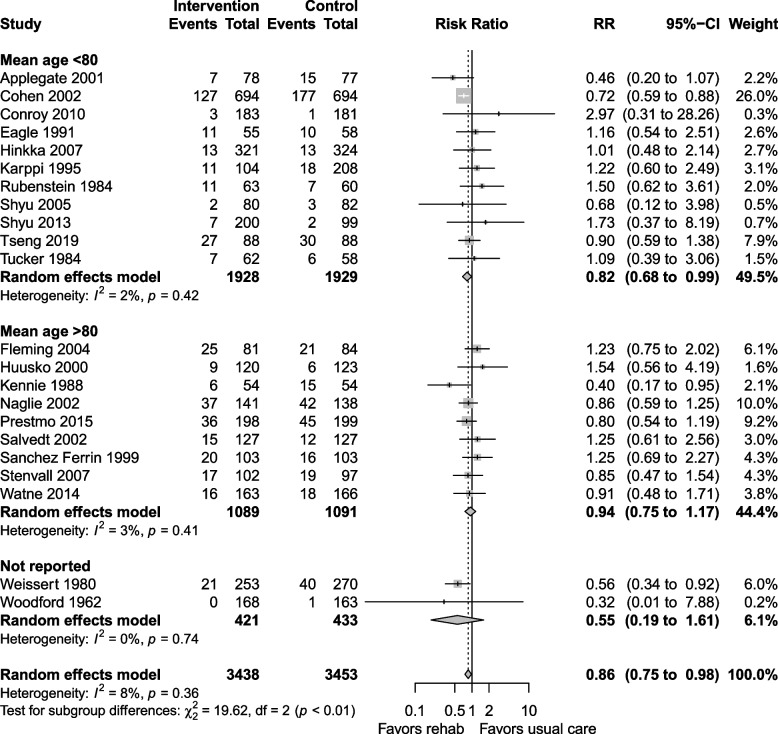
Subgroup analysis for LTCH admission at longest follow up: mean age < 80 vs. ≥ 80 years

Subgroup analysis of studies that followed patients for > 6 months showed a reduction in LTCH admission (RR 0.86, 0.73 to 1.02, I^2^ = 18%, Fig. 6), while studies that followed patients for ≤ 6 months did not have similar benefits (RR 0.96, 0.70 to 1.31, I^2^ = 0%). Studies including participants with mean age ≥ 80 years showed no difference in LTCH admission (RR 0.94, 0.75 to 1.17, I^2^ = 3%), while the subgroup with mean age < 80 years (Fig. 7) showed a decrease in LTCH admissions (RR 0.82, 0.68 to 0.99, I^2^ = 2%). Sensitivity analyses and LTCH admission at discharge are shown in Additional file 1: Appendix 4–5.

**Fig. 8:**
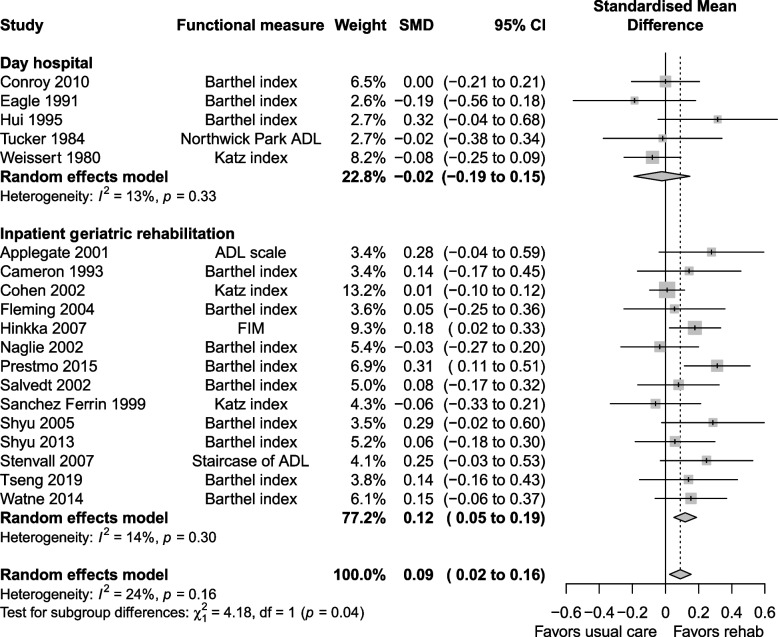
Functional status at longest follow up

**Fig. 9:**
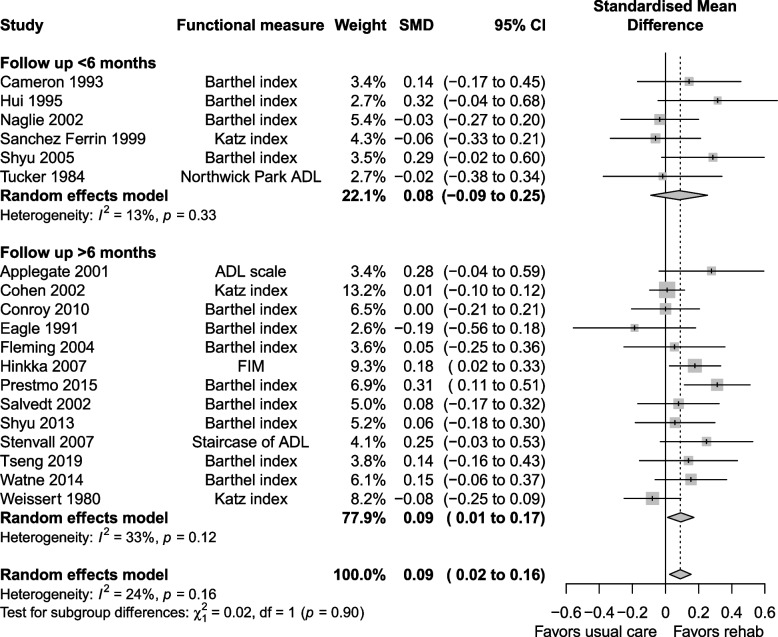
Subgroup analysis for functional status at longest follow up: follow up ≤ 6 months vs. > 6 months

**Fig. 10:**
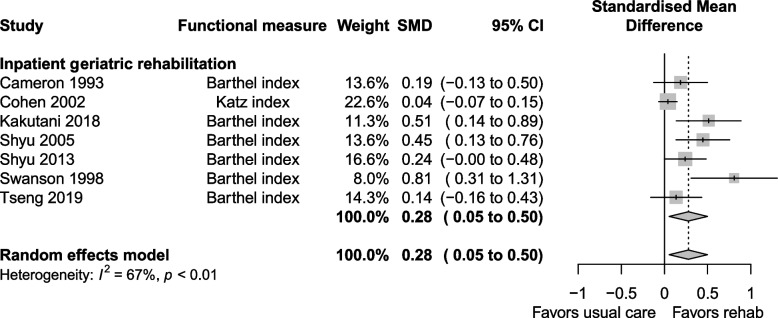
Functional status at discharge

The certainty of evidence for functional status was moderate, downgraded for risk of bias, imprecision and indirectness. Functional status at longest follow up was reported in 19 studies, with a SMD of 0.09 (0.02 to 0.16, I^2^ = 24%) favouring the geriatric rehabilitation group (Fig. 8). Subgroup analysis favoured the inpatient rehabilitation setting (SMD 0.12, 0.05 to 0.19, I^2^ = 14%) over day hospital (SMD −0.02, −0.19 to 0.15, I^2^ = 13%) for functional status. Subgroup analysis (Fig. 9) by follow up duration > 6 months (SMD 0.09, 0.01 to 0.17, I^2^ = 33%) vs. ≤ 6 months (SMD 0.08, −0.09 to 0.25, I^2^ = 13%) had similar estimates favouring geriatric rehabilitation. Functional status at discharge was reported in seven studies (Fig. 10), with a SMD of 0.28 (0.05 to 0.50, I^2^ = 67%). Heterogeneity was reduced when looking at subgroups by indication for rehabilitation, but not for other factors including mean age, cognitive status in eligibility criteria and team composition (Additional file 1: Appendix 6–7). For patients with hip fracture (only subgroup that had enough studies to pool), the SMD was 0.30 (0.03 to 0.56, I^2^ = 40%) at discharge. Estimates for functional status and other continuous outcomes are shown in Table 3 along with the corresponding MCID in natural units.

**Table 3:** Continuous outcomes in this meta-analysis with corresponding minimal clinically important difference (MCID). Each summary outcome measure was transformed back into natural units for comparison to the MCID [22]

Outcome (default measure)	Meta-analysis pooled estimate	Transformed to measure units	MCID
Functional status (Barthel index)	SMD 0.09 (0.02 to 0.16)	2.78 points (0.62 to 4.95)	9 to 18 points [39, 40]
Cognition (MMSE)	MD 0.97 points (0.35 to 1.60)	0.97 points (0.35 to 1.60)	1.4 to 2.0 points [41, 42]
Mood (GDS)	SMD −0.67 (−2.30 to 1.05)	−2.18 points (−7.48 to 3.41)	5 points [43]
Quality of life (SF-36)	SMD 0.09 (−0.11 to 0.28)	2.49 points (−3.04 to 7.74)	2 to 3 points [44]

*SMD* standardized mean difference, *MD* mean difference, *MMSE* mini-mental status examination, *GDS* geriatric depression scale, *SF-36* 36-item short form survey

### Secondary outcomes

#### Functional improvement, discharge home and length of stay

The certainty of evidence for functional improvement was moderate due to risk of bias. When defined as a binary outcome by individual study authors (11 studies), participants were more likely to have functional improvement at the longest follow up in the geriatric rehabilitation group (RR 1.37, 1.20 to 1.56, I^2^ = 20%) compared with usual care. The magnitude of effect in the inpatient rehabilitation setting was larger (RR 1.46, 1.24 to 1.71, I^2^ = 8%) than the effect in geriatric day hospital setting (RR 1.22, 0.84 to 1.77, I^2^ = 0). The risk difference for functional improvement was 0.13 (0.07 to 0.19), and the NNT was 7 (5 to 14). Subgroup analyses are shown in Additional file 1: Appendix 8–9.

The certainty of evidence for discharge home and remaining home at longest follow up was low due to risk of bias, imprecision and inconsistency. Discharge home was reported in 11 studies and was more common in the geriatric rehabilitation group (RR 1.26, 1.03 to 1.54, I^2^ = 63%). Heterogeneity was explored using subgroups of rehabilitation setting, age, attrition, indication for rehabilitation, cognitive status in eligibility criteria, and team composition, but effect estimates were similar in major subgroups. A meta-regression was done using age, sex, publication year, and proportion of patients who lived at home, but none of these variables explained the heterogeneity. The number of patients remaining home at longest follow up was reported by 10 studies. More patients remained home at longest follow up in the geriatric rehabilitation group (RR 1.15, 1.01 to 1.32, I^2^ = 53%), with heterogeneity explained by setting. The inpatient rehabilitation setting had a significant benefit (RR 1.21, 1.05 to 1.38, I^2^ = 40%) while day hospital did not (RR 0.97, 0.34 to 2.79, I^2^ = 36%). Other subgroups including mean age, attrition, follow up duration, indication for rehabilitation, cognitive status in eligibility criteria, and team composition did not explain the heterogeneity (Additional file 1: Appendix 10–11). The risk difference for patients remaining home was 0.09 (0.01 to 0.16) and the NNT was 11 (6 to 100).

The certainty of evidence for length of stay was low, downgraded for risk of bias, imprecision and inconsistency. Length of stay was reported in 18 studies, all conducted in the inpatient setting. There was a shorter stay (mean difference of 0.79 days [−4.74 to 3.17, I^2^ = 91%]) for the geriatric rehabilitation group. The heterogeneity was mainly from studies of patients with hip fractures. No subgroup or study characteristic explained the heterogeneity in length of stay data (Additional file 1: Appendix 12). A meta-regression was done using age, sex, publication year, and proportion from home, but heterogeneity was not reduced.

#### Cognition, mood, and quality of life

The certainty of evidence for cognition was moderate, downgraded due to imprecision. Cognition was reported in five studies at longest follow up, all of which reported MMSE and occurred in the inpatient setting (Additional file 1: Appendix 13). Cognition improved in the geriatric rehabilitation group by a mean difference of 0.97 points (0.35 to 1.60, I^2^ = 0%). The MCID for cognition is presented in Table 3.

The certainty of evidence for mood was low, downgraded due to risk of bias, imprecision, indirectness and inconsistency. Five studies reported mood at longest follow up using various scales. There were better mood scores in geriatric rehabilitation group compared with usual care (SMD −0.67, −2.30 to 1.05, I^2^ = 99%), but the confidence interval could not exclude worsening of mood scores. Some of the heterogeneity was explained by age, with studies reporting mean age < 80 years (SMD −0.29, −1.06 to 0.48, I^2^ = 0%) showing better mood scores than studies with mean age ≥ 80 years (SMD 0.13, −4.54 to 4.80, I^2^ = 94%). Other subgroups of follow up duration, indication for rehabilitation, cognitive status in eligibility criteria, and team composition did not explain heterogeneity (Additional file 1: Appendix 14). None of the studies reporting mood as an outcome included a psychiatrist or psychologist as part of the team. The MCID for mood is presented in Table 3.

The certainty of evidence for quality of life was low, downgraded for imprecision, indirectness and inconsistency. Quality of life was reported by one day hospital and five inpatient rehabilitation studies at longest follow up (Additional file 1: Appendix 15). Quality of life scores were better in the geriatric rehabilitation group (SMD 0.09, −0.11 to 0.28, I^2^ = 56%) compared to usual care, but the confidence intervals were not able to exclude worsening. Heterogeneity in scores could be partially explained by indication for rehabilitation, with better quality of life scores (SMD 0.21, −0.28 to 0.69, I^2^ = 59%) in the hip fracture studies compared with general geriatric rehabilitation (SMD 0.02, −0.30 to 0.34, I^2^ = 48%). Heterogeneity could be also be partially explained by the measure of quality of life with better scores in studies using SF-36 (SMD 0.21, −0.28 to 0.60, I^2^ = 56%) compared with other measures. The MCID for quality of life is presented in Table 3.

## Discussion

We determined that geriatric rehabilitation in the inpatient and day hospital settings were effective at reducing mortality and LTCH admission in older adults, with clinically important effects (NNT of 33 for mortality and 100 for LTCH admission). The certainty of evidence in the inpatient setting was higher than in the day hospital setting. Additional benefits included improved function and cognition, and increased number of patients remaining home. The overall findings suggest that older adults with rehabilitation goals should receive geriatric rehabilitation in inpatient and day hospital settings to prevent disability and death.

Previous systematic reviews reported on the efficacy of geriatric rehabilitation in different settings or population (Additional file 1: Appendix 16). In one of the previous systematic reviews of inpatient geriatric rehabilitation, similar results were found compared to our review for mortality, LTCH admission and functional improvement [4]. We included eight additional trials and excluded two trials from the previous review that did not meet our eligibility criteria [45, 46], contributing 1648 more patients to our review. One trial was excluded because the comparator group all received inpatient rehabilitation in hospital after being transferred from an inpatient geriatric unit [45], which is unlike the other usual care interventions. The other trial was excluded because it was more similar to an acute care of the elderly intervention instead of a post-acute rehabilitation intervention [46]. We also reported on clinically important outcomes that were not included in the previous review [4], such as functional status, cognition, remaining home, mood, and quality of life. In our subgroup analyses, we found that LTCH admission at longest follow up was lower in trials with a mean participant age of < 80 years, while the previous review reported no difference in the age subgroups [4]. We also found that the magnitude of effect for functional status and LTCH admission was larger in studies with a follow up duration of ≥ 6 months, unlike the previous review which found a smaller effect [4]. The optimal age for geriatric rehabilitation and duration of benefit are worth exploring in future research studies.

Another previous systematic review of inpatient rehabilitation for patients with hip fractures only [47] found that the composite outcome of death or LTCH admission was reduced in the intervention group (RR 0.88, 0.80 to 0.98), with a similar estimate in the mortality outcome (RR 0.91, 0.80 to 1.05). In our systematic review, we used a definition of geriatric rehabilitation similar to Bachmann et al. [4], which required that the intervention be designed for older adults and a multidisciplinary team be involved. Several studies included in the hip fracture systematic review did not meet this eligibility criteria [48–50] and were excluded from our review. We also excluded quasi-randomized [51] and non-randomized studies [52], which were included in the previous review of rehabilitation for patients with hip fracture. We further excluded one study that only included patients from LTCH [53]. Using our criteria for study inclusion, the pooled mortality benefit at longest follow up was RR 0.82 (0.73 to 0.92) for patients with hip fracture, which is similar to the previous review but a potentially larger effect.

For day hospital interventions, seven studies were excluded because they used an active comparator, such as domiciliary care or home-based rehabilitation, which we did not consider to be usual care. There was a trend towards benefit for the outcomes of mortality, LTCH admission, and functional improvement with geriatric day hospital, which should be further explored in trials with a usual care comparator and long term follow up. Another previous systematic review of day hospitals came to similar conclusions with less favourable estimates potentially due to inclusion of active intervention comparator groups [11].

The impact of geriatric rehabilitation on functional status does not appear clinically significant because the SMD does not meet the MCID. However, looking at the dichotomous outcome of functional improvement as defined by the individual study authors, the risk difference was 0.13 (0.07 to 0.19) and the NNT was 7, which is clinically significant. For cognition, our review found a mean difference of 0.97 points (0.35 to 1.60) higher in MMSE score for those in the geriatric rehabilitation group. This difference is below MCID of the MMSE tool when used in clinical trials of interventions for those with Alzheimer’s disease, which is estimated at 1.4 to 2.0 [41, 42]. The magnitude of cognitive improvement in geriatric rehabilitation is worth exploring in future studies, which is relevant to those with or at risk of cognitive impairment.

Despite incorporating PROGRESS-Plus criteria [19] in our data abstraction, almost no studies reported these equity characteristics, so the intersection between outcomes and social determinants of health could not be explored. Future studies should explore the impact of intersectionality on outcomes of geriatric rehabilitation and the implementation and sustainability of geriatric rehabilitation interventions in a more diverse population with varying degrees of frailty.

### Strengths/limitations

Our study had several strengths. We engaged with a patient partner and other experts in developing the protocol and outcomes. We comprehensively searched six databases and the grey literature for relevant studies. Determination of eligibility required a multidisciplinary intervention tailored to older adults, in keeping with best practices in designing a geriatric rehabilitation intervention [5]. We adhered to the PRISMA 2020 statement and incorporated PROGRESS-Plus criteria to ensure that equity characteristics were captured. We screened, abstracted, and appraised studies in duplicate.

Our study had several limitations. In some outcomes such as length of stay and discharge home, heterogeneity could not be fully explained. This was likely related to the complex design of interventions like geriatric rehabilitation, which may have different ‘dosages’ and ‘formulations’ [54]. We did not conduct a dose–response analysis given the heterogeneity of the interventions and lack of consistent information on ‘dosages’ [54]. We did not explore the impact of the different models of care in studies as these data were infrequently reported. We did not have individual patient data, which limits our ability to further explore outcomes by patient characteristics (such as their social determinants of health). We also did not use a tool like the Template for Intervention Description and Replication (TIDieR) checklist to determine the individual intervention components in each study [55].

## Conclusions

Geriatric rehabilitation in the inpatient and day hospital settings is effective in reducing mortality, preventing LTCH admissions, improving function in long term follow up, and may improve cognition. The certainty of evidence in the inpatient setting was higher than in the day hospital setting. Future studies should aim to optimize geriatric rehabilitation design and explore best practices in implementing this intervention in a diverse and aging population.

## Supplementary Information

Additional file 1: Appendix 1. Search strategy. Appendix 2. Mortality at longest follow up. Fig. S2.1 Subgroup analysis for mortality at longest follow up: indication for rehabilitation. Fig. S2.2 Subgroup analysis for mortality at longest follow up: mean age < 80 vs. ≥ 80 years. Fig. S2.3 Subgroup analysis for mortality at longest follow up: follow up ≤ 6 months vs. > 6 months. Fig. S2.4 Sensitivity analysis for mortality at longest follow up: Studies at low risk of bias from assignment to intervention. Fig. S2.5 Sensitivity analysis for mortality at longest follow up: Studies at low risk of bias from measurement of the outcome. Fig. S2.6 Subgroup analysis for mortality at longest follow up: cognitive status in eligibility criteria. Appendix 3. Mortality at discharge. Fig. S3.1 Subgroup analysis for mortality at discharge: indication for rehabilitation. Fig. S3.2 Subgroup analysis for mortality at discharge: mean age < 80 vs ≥ 80 years. Appendix 4. LTCH admission at longest follow up. Fig. S4.1 Subgroup analysis for LTCH admission at longest follow up: follow up ≤ 6 months vs. > 6 months. Fig. S4.2 Subgroup analysis for LTCH admission at longest follow up: mean age < 80 vs. ≥ 80 years. Fig. S4.3 Subgroup analysis for LTCH admission at longest follow up: indication for rehabilitation. Fig. S4.4 Sensitivity analysis: LTCH admission at longest follow up including only those at low risk of bias from assignment of intervention. Fig. S4.5 Sensitivity analysis: LTCH admission at longest follow up including only those at low risk of bias from measurement of outcome. Fig. S4.6 Subgroup analysis for LTCH admission at longest follow up: cognitive status in eligibility criteria. Appendix 5. LTCH admission at discharge. Appendix 6. Functional status at longest follow up. Fig. S6.1 Subgroup analysis for functional status at longest follow up: attrition < 10% vs. ≥ 10%. Fig. S6.2 Subgroup analysis for functional status at longest follow up: follow up ≤ 6 months vs. > 6 months. Fig. S6.3 Subgroup analysis for functional status at longest follow up: indication for rehabilitation. Fig. S6.4 Subgroup analysis for functional status at longest follow up: cognitive status in eligibility criteria. Appendix 7. Functional status at discharge. Fig. S7.1 Subgroup analysis for functional status at discharge: mean age < 80 vs. ≥ 80. Fig. S7.2 Subgroup analysis for functional status at discharge: indication for rehabilitation. Fig. S7.3 Subgroup analysis for functional status at discharge: geriatrician in team. Fig. S7.4 Subgroup analysis for functional status at discharge: OT in team. Fig. S7.5 Subgroup analysis for functional status at discharge: nurse in team. Fig. S7.6 Subgroup analysis for functional status at discharge: social worker in team. Fig. S7.7 Subgroup analysis for functional status at discharge: cognitive status in eligibility criteria. Appendix 8. Functional improvement (as defined by authors) at longest follow up. Fig. S8.1 Subgroup analysis for functional improvement at longest follow up: mean age < 80 vs. ≥ 80. Fig. S8.2 Subgroup analysis for functional improvement at longest follow up: attrition < 10% vs. ≥ 10%. Fig. S8.3 Subgroup analysis for functional improvement at longest follow up: follow up ≤ 6 months vs. > 6. Fig. S8.4 Subgroup analysis for functional improvement at longest follow up: indication for rehabilitation. Fig. S8.5 Subgroup analysis for functional improvement at longest follow up: cognitive status in eligibility criteria. Appendix 9. Functional improvement (as defined by authors) at discharge. Appendix 10. Discharge home. Fig. S10.1 Subgroup analysis for discharge home: mean age < 80 vs. > 80. Fig. S10.2 Subgroup analysis for discharge home: attrition < 10% vs. > 10%. Fig. S10.3 Subgroup analysis for discharge home: indication for rehabilitation. Fig. S10.4 Subgroup analysis for discharge home: geriatrician in team. Fig. S10.5 Subgroup analysis for discharge home: OT in team. Fig. S10.6 Subgroup analysis for discharge home: nurse in team. Fig. S10.7 Subgroup analysis for discharge home: social worker in team. Fig. S10.8 Subgroup analysis for discharge home: cognitive status in eligibility criteria. Appendix 11. Remaining home at longest follow up. Fig. S11.1 Subgroup analysis remaining home at longest follow up: mean age < 80 vs. ≥ 80. Fig. S11.2 Subgroup analysis remaining home at longest follow up: attrition < 10% vs. ≥ 10%. Fig. S11.3 Subgroup analysis remaining home at longest follow up: follow up < 6 vs. > 6 months. Fig. S11.4 Subgroup analysis remaining home at longest follow up: indication for rehabilitation. Fig. S11.5 Subgroup analysis remaining home at longest follow up: geriatrician in team. Fig. S11.6 Subgroup analysis remaining home at longest follow up: OT in team. Fig. S11.7 Subgroup analysis remaining home at longest follow up: nurse in team. Fig. S11.8 Subgroup analysis remaining home at longest follow up: social worker in team. Fig. S11.9 Subgroup analysis remaining home at longest follow up: cognitive status in eligibility criteria. Appendix 12. Length of stay in hospital. Fig. S12.1 Subgroup analysis for length of stay: mean age < 80 vs. > 80. Fig. S12.2 Subgroup analysis for length of stay: indication for rehabilitation. Fig. S12.3 Subgroup analysis for length of stay: geriatrician in team. Fig. S12.4 Subgroup analysis for length of stay: OT in team. Fig. S12.5 Subgroup analysis for length of stay: nurse in team. Fig. S12.6 Subgroup analysis for length of stay: social worker in team. Fig. S12.7 Subgroup analysis for length of stay: cognitive status in eligibility criteria. Appendix 13. Cognition at longest follow up. Appendix 14. Mood at longest follow up. Fig. S14.1 Subgroup analysis for mood: mean age < 80 vs. ≥ 80. Fig. S14.2 Subgroup analysis for mood: follow up < 6 vs. > 6 months. Fig. S14.3 Subgroup analysis for mood: indication for rehabilitation. Fig. S14.4 Subgroup analysis for mood: geriatrician in team. Fig. S14.5 Subgroup analysis for mood: OT in team. Fig. S14.6 Subgroup analysis for mood: nurse in team. Fig. S14.7 Subgroup analysis for mood: social worker in team. Fig. S14.8 Subgroup analysis for mood: cognitive status in eligibility criteria. Appendix 15. Quality of life at longest follow up. Fig. S15.1 Subgroup analysis for quality of life: mean age < 80 vs. ≥ 80. Fig. S15.2 Subgroup analysis for quality of life: follow up < 6 vs. > 6 months. Fig. S15.3 Subgroup analysis for quality of life: measure of quality of life. Fig. S15.4 Subgroup analysis for quality of life: indication for rehabilitation. Fig. S15.5 Subgroup analysis for quality of life: geriatrician in team. Fig. S15.6 Subgroup analysis for quality of life: OT in team. Fig. S15.7 Subgroup analysis for quality of life: nurse in team. Fig. S15.8 Subgroup analysis for quality of life: social worker in team. Fig. S15.9 Subgroup analysis for quality of life: cognitive status in eligibility criteria. Appendix 16. Supplementary table S1: Comparison of outcome estimates with other systematic reviews of geriatric rehabilitation.

## Data Availability

The datasets used and/or analysed during the current study are available from the corresponding author on reasonable request.

## Abbreviations

RR: Risk ratio
SMD: Standardized mean difference
MD: Mean difference
NNT: Number needed to treat
LTCH: Long-term care homes
MCID: Minimal clinically important difference
MMSE: Mini-mental status examination

## References

[1] World Health Organization. Rehabilitation in Health Systems. Geneva: World Health Organization; 2017. https://www.who.int/publications/i/item/9789241549974.

[2] World Health Organization. Rehabilitation 2030. WHO. 2021. https://www.who.int/initiatives/rehabilitation-2030. Accessed 28 Mar 2022.

[3] Vos T, Flaxman AD, Naghavi M, Lozano R, Michaud C, Ezzati M, et al. Years lived with disability (YLDs) for 1160 sequelae of 289 diseases and injuries 1990–2010: a systematic analysis for the Global Burden of Disease Study 2010. Lancet Lond Engl. 2012;380:2163–96. 10.1016/S0140-6736(12)61729-2 PMC6350784 23245607

[4] Bachmann S, Finger C, Huss A, Egger M, Stuck AE, Clough-Gorr KM. Inpatient rehabilitation specifically designed for geriatric patients: systematic review and meta-analysis of randomised controlled trials. BMJ. 2010;340:1230. 10.1136/bmj.c1718 PMC2857746 20406866

[5] van Balen R, Gordon AL, Schols JMGA, Drewes YM, Achterberg WP. What is geriatric rehabilitation and how should it be organized? A Delphi study aimed at reaching European consensus. Eur Geriatr Med. 2019;10:977–87. 34652774 10.1007/s41999-019-00244-7

[6] Hoenig H, Nusbaum N, Brummel-Smith K. Geriatric rehabilitation: state of the art. J Am Geriatr Soc. 1997;45:1371–81. 9361665 10.1111/j.1532-5415.1997.tb02939.x

[7] Kamenov K, Mills JA, Chatterji S, Cieza A. Needs and unmet needs for rehabilitation services: a scoping review. Disabil Rehabil. 2019;41:1227–37. 29303004 10.1080/09638288.2017.1422036

[8] Hirdes JP, Fries BE, Morris JN, Ikegami N, Zimmerman D, Dalby DM, et al. Home care quality indicators (HCQIs) based on the MDS-HC. Gerontologist. 2004;44:665–79. 15498842 10.1093/geront/44.5.665

[9] Preitschopf A, Holstege M, Ligthart A, Groen W, Burchell G, Pol M, et al. Effectiveness of outpatient geriatric rehabilitation after inpatient geriatric rehabilitation or hospitalisation: a systematic review and meta-analysis. Age Ageing. 2023;52:afac300. 36626320 10.1093/ageing/afac300 PMC9831263

[10] Black DA. The geriatric day hospital. Age Ageing. 2005;34:427–9. 16107449 10.1093/ageing/afi149

[11] Brown L, Forster A, Young J, Crocker T, Benham A, Langhorne P. Medical day hospital care for older people versus alternative forms of care. Cochrane Database Syst Rev. 2015;2015(6):CD001730. 26102196 10.1002/14651858.CD001730.pub3 PMC7068157

[12] Deeks J, Higgins J, Altman D. Chapter 10: Analysing data and undertaking meta-analyses. Cochrane Handbook for Systematic Reviews of Interventions version 6.3 (updated February 2022). 2022. https://training.cochrane.org/handbook/current/chapter-10. Accessed 12 Apr 2022.

[13] Page MJ, McKenzie JE, Bossuyt PM, Boutron I, Hoffmann TC, Mulrow CD, The PRISMA, et al. statement: an updated guideline for reporting systematic reviews. BMJ. 2020;2021:372. 10.1136/bmj.n71 PMC8005924 33782057

[14] Sabharwal S, Wilson H, Reilly P, Gupte CM. Heterogeneity of the definition of elderly age in current orthopaedic research. Springerplus. 2015;4:516. 26405636 10.1186/s40064-015-1307-x PMC4573966

[15] CADTH. Grey Matters: a practical tool for searching health-related grey literature | CADTH.ca. CADTH. 2018. https://www.cadth.ca/resources/finding-evidence/grey-matters. Accessed 20 Aug 2018.

[16] McGowan J, Sampson M, Salzwedel DM, Cogo E, Foerster V, Lefebvre C. PRESS Peer Review of Electronic Search Strategies: 2015 Guideline Statement. J Clin Epidemiol. 2016;75:40–6. 27005575 10.1016/j.jclinepi.2016.01.021

[17] Cochrane. Search filters | Cochrane Community. Cochrane Handbook for Systematic Reviews of Interventions. 2022. https://community.cochrane.org/search-filters. Accessed 21 Jun 2022.

[18] Cochrane Equity Methods. PROGRESS-Plus | Cochrane Equity. Cochrane. 2022. https://methods.cochrane.org/equity/projects/evidence-equity/progress-plus. Accessed 2 Jun 2022.

[19] O’Neill J, Tabish H, Welch V, Petticrew M, Pottie K, Clarke M, et al. Applying an equity lens to interventions: using PROGRESS ensures consideration of socially stratifying factors to illuminate inequities in health. J Clin Epidemiol. 2014;67:56–64. 24189091 10.1016/j.jclinepi.2013.08.005

[20] Chudyk AM, Stoddard R, Duhamel TA, Andreas B, Ashe MC, Daly-Cyr J, et al. Future directions for patient engagement in research: a participatory workshop with Canadian patient partners and academic researchers. Health Res Policy Syst. 2024;22:24. 38350974 10.1186/s12961-024-01106-w PMC10865599

[21] McGlothlin AE, Lewis RJ. Minimal clinically important difference: defining what really matters to patients. JAMA. 2014;312:1342–3. 25268441 10.1001/jama.2014.13128

[22] Murad MH, Wang Z, Chu H, Lin L. When continuous outcomes are measured using different scales: guide for meta-analysis and interpretation. BMJ. 2019;364:k4817. 30670455 10.1136/bmj.k4817 PMC6890471

[23] Higgins JP, Savović J, Page MJ, Elbers RG, Sterne JA. Chapter 8: Assessing risk of bias in a randomized trial. In: Cochrane Handbook for Systematic Reviews of Interventions. Version 6.4. Chichester: Wiley; 2023. p. 1.

[24] Shrier I, Boivin JF, Steele RJ, Platt RW, Furlan A, Kakuma R, et al. Should Meta-Analyses of Interventions Include Observational Studies in Addition to Randomized Controlled Trials? A Critical Examination of Underlying Principles. Am J Epidemiol. 2007;166:1203–9. 17712019 10.1093/aje/kwm189

[25] Tombaugh TN, McIntyre NJ. The mini-mental state examination: a comprehensive review. J Am Geriatr Soc. 1992;40:922–35. 1512391 10.1111/j.1532-5415.1992.tb01992.x

[26] Higgins JP, Green S. 12.5.4.1 Computing NNT from a risk difference. In: Cochrane Handbook for Systematic Reviews of Interventions version 5.1. 5.1. The Cochrane Collaboration; 2011.

[27] Deeks JJ, Altman DG, Bradburn MJ. Statistical Methods for Examining Heterogeneity and Combining Results from Several Studies in Meta-Analysis. In: Systematic Reviews in Health Care. London, UK: BMJ Publishing Group. p. 285–312.

[28] Jørgensen L, Paludan-Müller AS, Laursen DRT, Savović J, Boutron I, Sterne JAC, et al. Evaluation of the Cochrane tool for assessing risk of bias in randomized clinical trials: overview of published comments and analysis of user practice in Cochrane and non-Cochrane reviews. Syst Rev. 2016;5:80. 27160280 10.1186/s13643-016-0259-8 PMC4862216

[29] Riley RD, Higgins JPT, Deeks JJ. Interpretation of random effects meta-analyses. BMJ. 2011;342:d549. 21310794 10.1136/bmj.d549

[30] DerSimonian R, Laird N. Meta-analysis in clinical trials. Control Clin Trials. 1986;7:177–88. 3802833 10.1016/0197-2456(86)90046-2

[31] Higgins JPT, Thompson SG. Quantifying heterogeneity in a meta-analysis. Stat Med. 2002;21:1539–58. 12111919 10.1002/sim.1186

[32] Viechtbauer W. Conducting meta-analyses in R with the metafor. J Stat Softw. 2010;36:1–48.

[33] R Core Team. R: A language and environment for statistical computing. 2022.

[34] Egger M, Davey Smith G, Schneider M, Minder C. Bias in meta-analysis detected by a simple, graphical test. BMJ. 1997;315:629–34. 9310563 10.1136/bmj.315.7109.629 PMC2127453

[35] Guyatt GH, Oxman AD, Vist GE, Kunz R, Falck-Ytter Y, Alonso-Coello P, et al. GRADE: an emerging consensus on rating quality of evidence and strength of recommendations. BMJ. 2008;336:924–6. 18436948 10.1136/bmj.39489.470347.AD PMC2335261

[36] Watne LO, Torbergsen AC, Conroy S, Engedal K, Frihagen F, Hjorthaug GA, et al. The effect of a pre- and postoperative orthogeriatric service on cognitive function in patients with hip fracture: randomized controlled trial (Oslo Orthogeriatric Trial). BMC Med. 2014;12:63. 24735588 10.1186/1741-7015-12-63 PMC4022270

[37] Fordham R, Thompson R, Holmes J, Hodkinson C. A cost-benefit study of geriatric-orthopaedic management of patients with fractured neck of femur. Manuscript. York, UK.: University of York; 1986.

[38] Kakutani N, Fukushima A, Nakamura R, Mori S, Oikawa T, Ishimoto R, et al. Abstract 11606: critical pathway for elderly patients with heart failure based on physical activity reduces length of hospital stay. Circulation. 2014;130 suppl_2:A11606–A11606.

[39] Hsieh YW, Wang CH, Wu SC, Chen PC, Sheu CF, Hsieh CL. Establishing the minimal clinically important difference of the Barthel Index in stroke patients. Neurorehabil Neural Repair. 2007;21:233–8. 17351082 10.1177/1545968306294729

[40] Bouwstra H, Smit EB, Wattel EM, van der Wouden JC, Hertogh CMPM, Terluin B, et al. Measurement Properties of the Barthel Index in Geriatric Rehabilitation. J Am Med Dir Assoc. 2019;20:420–425.e1. 30448338 10.1016/j.jamda.2018.09.033

[41] Howard R, Phillips P, Johnson T, O’Brien J, Sheehan B, Lindesay J, et al. Determining the minimum clinically important differences for outcomes in the DOMINO trial. Int J Geriatr Psychiatry. 2011;26:812–7. 20848576 10.1002/gps.2607

[42] Watt JA, Veroniki AA, Tricco AC, Straus SE. Using a distribution-based approach and systematic review methods to derive minimum clinically important differences. BMC Med Res Methodol. 2021;21:41. 33637039 10.1186/s12874-021-01228-7 PMC7912575

[43] Quinten C, Kenis C, Decoster L, Debruyne PR, De Groof I, Focan C, et al. Determining clinically important differences in health-related quality of life in older patients with cancer undergoing chemotherapy or surgery. Qual Life Res Int J Qual Life Asp Treat Care Rehabil. 2019;28:663–76. 10.1007/s11136-018-2062-6 30511255

[44] Canadian Agency for Drugs and Technologies in Health. Validity of Outcome Measures. In: Clinical Review Report: Insulin Degludec (Tresiba). Ottawa: Canadian Agency for Drugs and Technologies in Health; 2017. p. 170.

[45] Young J, Green J, Forster A, Small N, Lowson K, Bogle S, et al. Postacute care for older people in community hospitals: a multicenter randomized, controlled trial. J Am Geriatr Soc. 2007;55:1995–2002. 17979957 10.1111/j.1532-5415.2007.01456.x

[46] White SJ, Powers JS, Knight JR, Harrell D, Varnell L, Vaughn C, et al. Effectiveness of an inpatient geriatric service in a university hospital. J Tenn Med Assoc. 1994;87(10):425–8. 7990452

[47] Handoll HHG, Cameron ID, Mak JCS, Panagoda CE, Finnegan TP. Multidisciplinary rehabilitation for older people with hip fractures. Cochrane Database Syst Rev. 2021;2021:CD007125. 10.1002/14651858.CD007125.pub3 PMC8586844 34766330

[48] Galvard H, Samuelsson SM. Orthopedic or geriatric rehabilitation of hip fracture patients: a prospective, randomized, clinically controlled study in Malmo, Sweden. Aging (Milan, Italy). 1995;7:11–6. 7599241 10.1007/BF03324284

[49] Marcantonio ER, Flacker JM, Wright RJ, Resnick NM. Reducing delirium after hip fracture: a randomized trial. J Am Geriatr Soc. 2001;49:516–22. 11380742 10.1046/j.1532-5415.2001.49108.x

[50] Chong TW, Chan G, Feng L, Goh S, Hew A, Ng TP, et al. Integrated care pathway for hip fractures in a subacute rehabilitation setting. Ann Acad Med Singapore. 2013;42:579–84. 24356654

[51] Jette AM, Harris BA, Cleary PD, Campion EW. Functional recovery after hip fracture. Arch Phys Med Rehabil. 1987;68:735–40. 3662784

[52] Baroni M, Serra R, Boccardi V, Ercolani S, Zengarini E, Casucci P, et al. The orthogeriatric comanagement improves clinical outcomes of hip fracture in older adults. Osteoporos Int J Establ Result Coop Eur Found Osteoporos Natl Osteoporos Found USA. 2019;30:907–16. 10.1007/s00198-019-04858-2 30715561

[53] Uy C, Kurrle SE, Cameron ID. Inpatient multidisciplinary rehabilitation after hip fracture for residents of nursing homes: a randomised trial. Australas J Ageing. 2008;27:43–4. 18713215 10.1111/j.1741-6612.2007.00277.x

[54] Veroniki AA, Soobiah C, Nincic V, Lai Y, Rios P, MacDonald H, et al. Efficacy of sustained knowledge translation (KT) interventions in chronic disease management in older adults: systematic review and meta-analysis of complex interventions. BMC Med. 2023;21:269. 37488589 10.1186/s12916-023-02966-9 PMC10367354

[55] Hoffmann TC, Glasziou PP, Boutron I, Milne R, Perera R, Moher D, et al. Better reporting of interventions: template for intervention description and replication (TIDieR) checklist and guide. BMJ. 2014;348:g1687. 24609605 10.1136/bmj.g1687

