# Supplementary material for: Effectiveness of geriatric rehabilitation in inpatient and day hospital settings: a systematic review and meta-analysis
Source: BMC Med. 2024 Nov 22;22:551. doi: 10.1186/s12916-024-03764-7 (PMC11583748; doi:10.1186/s12916-024-03764-7)
Supplement: Supplementary file 1 — Additional file 1: Appendix 1. Search strategy. Appendix 2. Mortality at longest follow up. Fig. S2.1 Subgroup analysis for mortality at longest follow up: indication for rehabilitation. Fig. S2.2 Subgroup analysis for mortality at longest follow up: mean age < 80 vs. ≥ 80 years. Fig. S2.3 Subgroup analysis for mortality at longest follow up: follow up ≤ 6 months vs. > 6 months. Fig. S2.4 Sensitivity analysis for mortality at longest follow up: Studies at low risk of bias from assignment to intervention. Fig. S2.5 Sensitivity analysis for mortality at longest follow up: Studies at low risk of bias from measurement of the outcome. Fig. S2.6 Subgroup analysis for mortality at longest follow up: cognitive status in eligibility criteria. Appendix 3. Mortality at discharge. Fig. S3.1 Subgroup analysis for mortality at discharge: indication for rehabilitation. Fig. S3.2 Subgroup analysis for mortality at discharge: mean age < 80 vs ≥ 80 years. Appendix 4. LTCH admission at longest follow up. Fig. S4.1 Subgroup analysis for LTCH admission at longest follow up: follow up ≤ 6 months vs. > 6 months. Fig. S4.2 Subgroup analysis for LTCH admission at longest follow up: mean age < 80 vs. ≥ 80 years. Fig. S4.3 Subgroup analysis for LTCH admission at longest follow up: indication for rehabilitation. Fig. S4.4 Sensitivity analysis: LTCH admission at longest follow up including only those at low risk of bias from assignment of intervention. Fig. S4.5 Sensitivity analysis: LTCH admission at longest follow up including only those at low risk of bias from measurement of outcome. Fig. S4.6 Subgroup analysis for LTCH admission at longest follow up: cognitive status in eligibility criteria. Appendix 5. LTCH admission at discharge. Appendix 6. Functional status at longest follow up. Fig. S6.1 Subgroup analysis for functional status at longest follow up: attrition < 10% vs. ≥ 10%. Fig. S6.2 Subgroup analysis for functional status at longest follow up: follow up ≤ 6 months vs. > 6 months. Fi [file 12916_2024_3764_MOESM1_ESM.docx]

Appendices for geriatric rehabilitation systematic review and meta-analysis

[Appendix 1. Search strategy 4](#_Toc176629350)

[Appendix 2. Mortality at longest follow up 9](#_Toc176629351)

[2.1 Subgroup analysis for mortality at longest follow up: indication for rehabilitation 10](#_Toc176629352)

[2.2 Subgroup analysis for mortality at longest follow up: mean age <80 vs. ≥80 years 11](#_Toc176629353)

[2.3 Subgroup analysis for mortality at longest follow up: follow up ≤6 months vs. >6 months 12](#_Toc176629354)

[2.4 Sensitivity analysis for mortality at longest follow up: Studies at low risk of bias from assignment to intervention 13](#_Toc176629355)

[2.5 Sensitivity analysis for mortality at longest follow up: Studies at low risk of bias from measurement of the outcome 13](#_Toc176629356)

[2.6 Subgroup analysis for mortality at longest follow up: cognitive status in eligibility criteria 14](#_Toc176629357)

[Appendix 3. Mortality at discharge 15](#_Toc176629358)

[3.1 Subgroup analysis for mortality at discharge: indication for rehabilitation 16](#_Toc176629359)

[3.2 Subgroup analysis for mortality at discharge: mean age <80 vs ≥80 years 17](#_Toc176629360)

[Appendix 4. LTCH admission at longest follow up 18](#_Toc176629361)

[4.1 Subgroup analysis for LTCH admission at longest follow up: follow up ≤6 months vs. > 6 months 19](#_Toc176629362)

[4.2 Subgroup analysis for LTCH admission at longest follow up: mean age <80 vs. ≥80 years 20](#_Toc176629363)

[4.3 Subgroup analysis for LTCH admission at longest follow up: indication for rehabilitation 21](#_Toc176629364)

[4.4 Sensitivity analysis: LTCH admission at longest follow up including only those at low risk of bias from assignment of intervention. 22](#_Toc176629365)

[4.5 Sensitivity analysis: LTCH admission at longest follow up including only those at low risk of bias from measurement of outcome. 23](#_Toc176629366)

[4.6 Subgroup analysis for LTCH admission at longest follow up: cognitive status in eligibility criteria 24](#_Toc176629367)

[Appendix 5. LTCH admission at discharge 25](#_Toc176629368)

[Appendix 6. Functional status at longest follow up 26](#_Toc176629369)

[6.1 Subgroup analysis for functional status at longest follow up: attrition <10% vs. ≥10% 27](#_Toc176629370)

[6.2 Subgroup analysis for functional status at longest follow up: follow up ≤6 months vs. >6 months 28](#_Toc176629371)

[6.3 Subgroup analysis for functional status at longest follow up: indication for rehabilitation 29](#_Toc176629372)

[6.4 Subgroup analysis for functional status at longest follow up: cognitive status in eligibility criteria 30](#_Toc176629373)

[Appendix 7. Functional status at discharge 31](#_Toc176629374)

[7.1 Subgroup analysis for functional status at discharge: mean age <80 vs. ≥80 31](#_Toc176629375)

[7.2 Subgroup analysis for functional status at discharge: indication for rehabilitation 32](#_Toc176629376)

[7.3 Subgroup analysis for functional status at discharge: geriatrician in team 32](#_Toc176629377)

[7.4 Subgroup analysis for functional status at discharge: OT in team 33](#_Toc176629378)

[7.5 Subgroup analysis for functional status at discharge: nurse in team 33](#_Toc176629379)

[7.6 Subgroup analysis for functional status at discharge: social worker in team 34](#_Toc176629380)

[7.7 Subgroup analysis for functional status at discharge: cognitive status in eligibility criteria 34](#_Toc176629381)

[Appendix 8. Functional improvement (as defined by authors) at longest follow up 35](#_Toc176629382)

[8.1 Subgroup analysis for functional improvement at longest follow up: mean age <80 vs. ≥80 36](#_Toc176629383)

[8.2 Subgroup analysis for functional improvement at longest follow up: attrition <10% vs. ≥10% 37](#_Toc176629384)

[8.3 Subgroup analysis for functional improvement at longest follow up: follow up ≤6 months vs. >6 38](#_Toc176629385)

[8.4 Subgroup analysis for functional improvement at longest follow up: indication for rehabilitation 39](#_Toc176629386)

[8.5 Subgroup analysis for functional improvement at longest follow up: cognitive status in eligibility criteria 40](#_Toc176629387)

[Appendix 9. Functional improvement (as defined by authors) at discharge 41](#_Toc176629388)

[Appendix 10. Discharge home 41](#_Toc176629389)

[10.1 Subgroup analysis for discharge home: mean age <80 vs. >80 42](#_Toc176629390)

[10.2 Subgroup analysis for discharge home: attrition <10% vs. >10% 43](#_Toc176629391)

[10.3 Subgroup analysis for discharge home: indication for rehabilitation 44](#_Toc176629392)

[10.4 Subgroup analysis for discharge home: geriatrician in team 44](#_Toc176629393)

[10.5 Subgroup analysis for discharge home: OT in team 45](#_Toc176629394)

[10.6 Subgroup analysis for discharge home: nurse in team 45](#_Toc176629395)

[10.7 Subgroup analysis for discharge home: social worker in team 46](#_Toc176629396)

[10.8 Subgroup analysis for discharge home: cognitive status in eligibility criteria 46](#_Toc176629397)

[Appendix 11. Remaining home at longest follow up 47](#_Toc176629398)

[11.1 Subgroup analysis remaining home at longest follow up: mean age <80 vs. ≥80 47](#_Toc176629399)

[11.2 Subgroup analysis remaining home at longest follow up: attrition <10% vs. ≥10% 48](#_Toc176629400)

[11.3 Subgroup analysis remaining home at longest follow up: follow up <6 vs. >6 months 49](#_Toc176629401)

[11.4 Subgroup analysis remaining home at longest follow up: indication for rehabilitation 50](#_Toc176629402)

[11.5 Subgroup analysis remaining home at longest follow up: geriatrician in team 50](#_Toc176629403)

[11.6 Subgroup analysis remaining home at longest follow up: OT in team 51](#_Toc176629404)

[11.7 Subgroup analysis remaining home at longest follow up: nurse in team 51](#_Toc176629405)

[11.8 Subgroup analysis remaining home at longest follow up: social worker in team 52](#_Toc176629406)

[11.9 Subgroup analysis remaining home at longest follow up: cognitive status in eligibility criteria 53](#_Toc176629407)

[Appendix 12. Length of stay in hospital 54](#_Toc176629408)

[12.1 Subgroup analysis for length of stay: mean age <80 vs. >80 55](#_Toc176629409)

[12.2 Subgroup analysis for length of stay: indication for rehabilitation 56](#_Toc176629410)

[12.3 Subgroup analysis for length of stay: geriatrician in team 57](#_Toc176629411)

[12.4 Subgroup analysis for length of stay: OT in team 57](#_Toc176629412)

[12.5 Subgroup analysis for length of stay: nurse in team 58](#_Toc176629413)

[12.6 Subgroup analysis for length of stay: social worker in team 58](#_Toc176629414)

[12.7 Subgroup analysis for length of stay: cognitive status in eligibility criteria 59](#_Toc176629415)

[Appendix 13. Cognition at longest follow up 60](#_Toc176629416)

[Appendix 14. Mood at longest follow up 61](#_Toc176629417)

[14.1 Subgroup analysis for mood: mean age <80 vs. ≥80 61](#_Toc176629418)

[14.2 Subgroup analysis for mood: follow up <6 vs. >6 months 62](#_Toc176629419)

[14.3 Subgroup analysis for mood: indication for rehabilitation 62](#_Toc176629420)

[14.4 Subgroup analysis for mood: geriatrician in team 63](#_Toc176629421)

[14.5 Subgroup analysis for mood: OT in team 63](#_Toc176629422)

[14.6 Subgroup analysis for mood: nurse in team 64](#_Toc176629423)

[14.7 Subgroup analysis for mood: social worker in team 64](#_Toc176629424)

[14.8 Subgroup analysis for mood: cognitive status in eligibility criteria 65](#_Toc176629425)

[Appendix 15. Quality of life at longest follow up 66](#_Toc176629426)

[15.1 Subgroup analysis for quality of life: mean age <80 vs. ≥80 66](#_Toc176629427)

[15.2 Subgroup analysis for quality of life: follow up <6 vs. >6 months 67](#_Toc176629428)

[15.3 Subgroup analysis for quality of life: measure of quality of life 67](#_Toc176629429)

[15.4 Subgroup analysis for quality of life: indication for rehabilitation 68](#_Toc176629430)

[15.5 Subgroup analysis for quality of life: geriatrician in team 68](#_Toc176629431)

[15.6 Subgroup analysis for quality of life: OT in team 69](#_Toc176629432)

[15.7 Subgroup analysis for quality of life: nurse in team 69](#_Toc176629433)

[15.8 Subgroup analysis for quality of life: social worker in team 70](#_Toc176629434)

[15.9 Subgroup analysis for quality of life: cognitive status in eligibility criteria 70](#_Toc176629435)

[Appendix 16. Supplementary table S1: Comparison of outcome estimates with other systematic reviews of geriatric rehabilitation. 71](#_Toc176629436)

## Appendix 1. Search strategy

Search strategy

**Database: Ovid MEDLINE(R) ALL <1946 to September 21, 2022>**
--------------------------------------------------------------------------------
1     (((inpatient or in-patient) adj2 "geriatric rehab*") or "geriatric day hospital" or ((aged or geriatric* or elder* or older or ageing or aging or senior) adj5 ("inpatient rehab*" or "in-patient rehab*"))).tw,kf. (477)
2     exp Rehabilitation/ or exp Physical Therapy Modalities/ or *disability evaluation/ or *postoperative care/mt or *functional status/ or exp *postural balance/ or *activities of daily living/ or *recovery of function/ (393518)
3     (Rehab* or "Motion Therapy" or mobilization or mobilisation or mobility or "physical function*" or "physical therap*" or physiotherapy* or exercise* or "strength train*" or "weight bearing" or "resistance train*" or "resistance program" or "functional recovery" or falls or instability or reablement or reable or reabled or "occupational therap*" or "early ambulation" or "therapeutic recreation" or "recreation therap*").tw,kf. (1014576)
4     2 or 3 (1252958)

5     *Inpatients/ or *Outpatient Clinics, Hospital/ or *Hospital Units/ or Day Care, Medical/ (31987)
6     ("day hospital" or "inpatient" or "in-patient" or "rehabilitation unit" or "rehabilitation center" or "rehabilitation centre" or "geriatric rehab*" or "intermediate care facility*" or "department of rehabilitation" or "rehab ward" or "community hospital" or "rehabilitation hospital" or (day care adj2 hospital*)).tw,kf. (192380)
7     5 or 6 (215950)
8     4 and 7 (31941)

9     limit 8 to "all aged (65 and over)" (12588)
10     (aged or geriatric* or elder* or older or ageing or aging or senior).tw,kf. or aging/ or frail elderly/ or geriatrics/ or aged/ (4350973)
11     8 and 10 (14425)
12     1 or 9 or 11 (14583)
13     (randomized controlled trial or controlled clinical trial).pt. or (randomized or placebo or randomly).ab. or clinical trials as topic/ or trial.ti. (1474389)
14     12 and 13 (2095)
15     (animals/ not humans/) or comment/ or editorial/ or hi.fs. or case report.mp. (7025871)
16     14 not 15 (2089)

**Notes:**

Line 1: General textword search - *This concept is poorly indexed, but I think the majority of your included studies will be from this set of 477 citation.

Line 4: Rehabilitation terms

Line 9: inpatient / day care terms (this is a difficult part of the search)

Line 12: Rehab and day care terms limited to geriatric plus line 1 (general tx search)

Line 13: Cochrane RCT filter

**Filters used:**

- Cochrane RCT filter (sensitivity- and precision-maximizing version)
- Age filter used (and modified): <https://extranet.santecom.qc.ca/wiki/!biblio3s/doku.php?id=concepts:personnes_agees>

**Database: EBM Reviews - Cochrane Central Register of Controlled Trials <August 2022>**
--------------------------------------------------------------------------------
1     (((inpatient or in-patient) adj2 "geriatric rehab*") or "geriatric day hospital" or ((aged or geriatric* or elder* or older or ageing or aging or senior) adj5 ("inpatient rehab*" or "in-patient rehab*"))).tw. (92)
2     (Rehab* or "Motion Therapy" or mobilization or mobilisation or mobility or "physical function*" or "physical therap*" or physiotherapy* or exercise* or "strength train*" or "weight bearing" or "resistance train*" or "resistance program" or "functional recovery" or falls or instability or reablement or reable or reabled or "occupational therap*" or "early ambulation" or "therapeutic recreation" or "recreation therap*").tw. (184317)
3     ("day hospital" or "inpatient" or "in-patient" or "rehabilitation unit" or "rehabilitation center" or "rehabilitation centre" or "geriatric rehab*" or "intermediate care facility*" or "department of rehabilitation" or "rehab ward" or "community hospital" or "rehabilitation hospital" or (day care adj2 hospital*)).tw. (28422)
4     (geriatric* or elder* or "older adult" or ageing or aging or senior).tw. (68875)
5     2 and 3 and 4 (743)
6     1 or 5 (777)

**Note:**

- Design is not needed.

**Database: Embase Classic+Embase <1947 to 2022 September 21>**
--------------------------------------------------------------------------------
1     (((inpatient or in-patient) adj2 "geriatric rehab*") or "geriatric day hospital" or ((aged or geriatric* or elder* or older or ageing or aging or senior) adj5 ("inpatient rehab*" or "in-patient rehab*"))).tw,kf. (737)
2     Rehabilitation/ or rehabilitation care/ or exp physiotherapy/ or occupational therapy/ or *Disability/ or *Postoperative Care/ or functional assessment/ or functional training/ or heart rehabilitation/ or muscle training/ or pulmonary rehabilitation/ or *daily life activity/ or bibliotherapy/ or recreational therapy/ (396014)
3     (Rehab* or "Motion Therapy" or mobilization or mobilisation or mobility or "physical function*" or "physical therap*" or physiotherapy* or exercise* or "strength train*" or "weight bearing" or "resistance train*" or "resistance program" or "functional recovery" or falls or instability or reablement or reable or reabled or "occupational therap*" or "early ambulation" or "therapeutic recreation" or "recreation therap*").tw. (1356433)
4     2 or 3 (1537704)
5     hospital patient/ or outpatient department/ or institutionalization/ or day care/ or geriatric hospital/ (336777)
6     ("day hospital" or "inpatient" or "in-patient" or "rehabilitation unit" or "rehabilitation center" or "rehabilitation centre" or "geriatric rehab*" or "intermediate care facility*" or "department of rehabilitation" or "rehab ward" or "community hospital" or "rehabilitation hospital" or (day care adj2 hospital*)).tw. (335487)
7     5 or 6 (538352)
8     4 and 7 (64584)
9     limit 8 to aged <65+ years> (17888)
10     Aged/ or frail elderly/ or geriatrics/ or very elderly/ or aging/ or (geriatric* or elder* or old* or ageing or aging).tw. (5840045)
11     8 and 10 (26683)
12     9 or 11 (26683)
13     12 or 1 (26896)
14     Randomized controlled trial/ or Controlled clinical study/ or random$.ti,ab. or randomization/ or intermethod comparison/ or placebo.ti,ab. or (compare or compared or comparison).ti. or ((evaluated or evaluate or evaluating or assessed or assess) and (compare or compared or comparing or comparison)).ab. or (open adj label).ti,ab. or ((double or single or doubly or singly) adj (blind or blinded or blindly)).ti,ab. or double blind procedure/ or parallel group$1.ti,ab. or (crossover or cross over).ti,ab. or ((assign$ or match or matched or allocation) adj5 (alternate or group$1 or intervention$1 or patient$1 or subject$1 or participant$1)).ti,ab. or (assigned or allocated).ti,ab. or (controlled adj7 (study or design or trial)).ti,ab. or (volunteer or volunteers).ti,ab. or human experiment/ or trial.ti. (5992605)
15     ((random$ adj sampl$ adj7 ("cross section$" or questionnaire$1 or survey$ or database$1)).ti,ab. not (comparative study/ or controlled study/ or randomi?ed controlled.ti,ab. or randomly assigned.ti,ab.)) or (Cross-sectional study/ not (randomized controlled trial/ or controlled clinical study/ or controlled study/ or randomi?ed controlled.ti,ab. or control group$1.ti,ab.)) or (((case adj control$) and random$) not randomi?ed controlled).ti,ab. or (Systematic review not (trial or study)).ti. or (nonrandom$ not random$).ti,ab. or "Random field$".ti,ab. or (random cluster adj3 sampl$).ti,ab. or ((review.ab. and [review.pt](http://review.pt).) not trial.ti.) or ("we searched".ab. and (review.ti. or [review.pt](http://review.pt).)) or "update review".ab. or (databases adj4 searched).ab. or ((rat or rats or mouse or mice or swine or porcine or murine or sheep or lambs or pigs or piglets or rabbit or rabbits or cat or cats or dog or dogs or cattle or bovine or monkey or monkeys or trout or marmoset$1).ti. and animal experiment/) or (Animal experiment/ not (human experiment/ or human/)) (4057898)
16     14 not 15 (5311757)
17     13 and 16 (6474)
18     comment/ or editorial/ or case report/ (3587005)
19     17 not 18 (6323)
20     (exp animal/ or animal experiment/ or nonhuman/) not (exp human/ or human experiment/) (7781998)
21     19 not 20 (6321)
22     limit 21 to yr=2011-current (4163)
23     limit 21 to yr=2016-current (2717)

**Filters used:**

- **RCT filter (**adapted from the Cochrane HHS for Embase) - <https://sites.google.com/a/york.ac.uk/issg-search-filters-resource/home/rcts/embase-rct-filter>

**Database: APA PsycInfo <1806 to September Week 1 2022>**
--------------------------------------------------------------------------------
1     (((inpatient or in-patient) adj2 "geriatric rehab*") or "geriatric day hospital" or ((aged or geriatric* or elder* or older or ageing or aging or senior) adj5 ("inpatient rehab*" or "in-patient rehab*"))).tw. (139)
2     exp rehabilitation/ or physical therapy/ or Disability Evaluation/ or functional status/ or ability level/ or daily activities/ or "Activities of Daily Living"/ (70139)
3     (Rehab* or "Motion Therapy" or mobilization or mobilisation or mobility or "physical function*" or "physical therap*" or physiotherapy* or exercise* or "strength train*" or "weight bearing" or "resistance train*" or "resistance program" or "functional recovery" or falls or instability or reablement or reable or reabled or "occupational therap*" or "early ambulation" or "therapeutic recreation" or "recreation therap*").tw. (212762)
4     hospitalized patients/ or outpatient treatment/ or adult day care/ or hospitalization/ or geriatric patients/ (42237)
5     ("day hospital" or "inpatient" or "in-patient" or "rehabilitation unit" or "rehabilitation center" or "rehabilitation centre" or "geriatric rehab*" or "intermediate care facility*" or "department of rehabilitation" or "rehab ward" or "community hospital" or "rehabilitation hospital" or (day care adj2 hospital*)).tw. (48345)
6     2 or 3 (239574)
7     4 or 5 (82842)
8     6 and 7 (12191)
9     limit 8 to "380 aged <age 65 yrs and older>" (4271)
10     (aged or geriatric* or elder* or older or ageing or aging or senior).tw. or aging/ or frail elderly/ or geriatrics/ or aged/ (516932)
11     8 and 10 (4097)
12     1 or 9 or 11 (5520)
13     (randomized controlled trial or controlled clinical trial).pt. or (randomized or placebo or randomly).ab. or clinical trials as topic/ or trial.ti. (190428)
14     12 and 13 (572)
15     (animals/ not humans/) or comment/ or editorial/ or case report/ (30688)
16     14 not 15 (572)

**PEDRO -** [**https://pedro.org.au**](https://pedro.org.au)

*Limit by method to: clinical trial

Search for each of the following lines in the “Abstract and title” field only:

- geriatric day hospital (30 records)
- elderly day hospital (41 records)
- inpatient geriatric (45 records)
- inpatient elderly (36 records)
- in-patient geriatric (63 records)
- inpatient elderly (171 records)
- hospitalized geriatric (179 records)

Note:

- There is no need to add any rehab terms, as all articles will technically have a rehab term for inclusion.

**AgeLine (EBSCO)**

*Searching using keywords using the “All textword” fields

Line 1: aged or geriatric* or elder* or older adult or ageing or aging or senior

Line 2: Rehab* or Motion Therapy or functional status physiotherapy* or mobility or mobilization or mobilisation or mobility or physical function* or physical therap* or physiotherapy* or exercise* or strength train* or weight bearing or resistance train* or resistance program or functional recovery or falls or instability or reablement or reable or reabled or reabling or Occupational Therap*

Line 3: day hospital or inpatient or in-patient or rehabilitation unit or rehabilitation center or rehabilitation centre or geriatric rehab* or intermediate care facility* or department of rehabilitation or rehab ward or community hospital or rehabilitation hospital

Line 4: randomized controlled trial* or randomised control trial* or RCT or RCTs

Results = 711

## Appendix 2. Mortality at longest follow up

### 2.1 Subgroup analysis for mortality at longest follow up: indication for rehabilitation

### 2.2 Subgroup analysis for mortality at longest follow up: mean age <80 vs. ≥80 years

### 2.3 Subgroup analysis for mortality at longest follow up: follow up ≤6 months vs. >6 months

### 2.4 Sensitivity analysis for mortality at longest follow up: Studies at low risk of bias from assignment to intervention

### 2.5 Sensitivity analysis for mortality at longest follow up: Studies at low risk of bias from measurement of the outcome

### 2.6 Subgroup analysis for mortality at longest follow up: cognitive status in eligibility criteria

Subgroups: (i) included patients with dementia of any severity, (ii) included patients with mild to moderate dementia (excluded severe dementia), (iii) excluded patients with dementia, and (iv) not reported. 95% CI = 95% confidence interval, RR = risk ratio

## Appendix 3. Mortality at discharge

### 3.1 Subgroup analysis for mortality at discharge: indication for rehabilitation

### 3.2 Subgroup analysis for mortality at discharge: mean age <80 vs ≥80 years

## Appendix 4. LTCH admission at longest follow up

### 4.1 Subgroup analysis for LTCH admission at longest follow up: follow up ≤6 months vs. > 6 months

### 4.2 Subgroup analysis for LTCH admission at longest follow up: mean age <80 vs. ≥80 years

### 4.3 Subgroup analysis for LTCH admission at longest follow up: indication for rehabilitation

### 4.4 Sensitivity analysis: LTCH admission at longest follow up including only those at low risk of bias from assignment of intervention.

### 4.5 Sensitivity analysis: LTCH admission at longest follow up including only those at low risk of bias from measurement of outcome.

### 4.6 Subgroup analysis for LTCH admission at longest follow up: cognitive status in eligibility criteria

Subgroups: (i) included patients with dementia of any severity, (ii) included patients with mild to moderate dementia (excluded severe dementia), (iii) excluded patients with dementia, and (iv) not reported. 95% CI = 95% confidence interval, RR = risk ratio

## Appendix 5. LTCH admission at discharge

## Appendix 6. Functional status at longest follow up

### 6.1 Subgroup analysis for functional status at longest follow up: attrition <10% vs. ≥10%

### 6.2 Subgroup analysis for functional status at longest follow up: follow up ≤6 months vs. >6 months

### 6.3 Subgroup analysis for functional status at longest follow up: indication for rehabilitation

### 6.4 Subgroup analysis for functional status at longest follow up: cognitive status in eligibility criteria

Subgroups: (i) included patients with dementia of any severity, (ii) included patients with mild to moderate dementia (excluded severe dementia), (iii) excluded patients with dementia, and (iv) not reported.

## Appendix 7. Functional status at discharge

### 7.1 Subgroup analysis for functional status at discharge: mean age <80 vs. ≥80

### 7.2 Subgroup analysis for functional status at discharge: indication for rehabilitation

### 7.3 Subgroup analysis for functional status at discharge: geriatrician in team

### 7.4 Subgroup analysis for functional status at discharge: OT in team

### 7.5 Subgroup analysis for functional status at discharge: nurse in team

### 7.6 Subgroup analysis for functional status at discharge: social worker in team

### 7.7 Subgroup analysis for functional status at discharge: cognitive status in eligibility criteria

## Appendix 8. Functional improvement (as defined by authors) at longest follow up

### 8.1 Subgroup analysis for functional improvement at longest follow up: mean age <80 vs. ≥80

### 8.2 Subgroup analysis for functional improvement at longest follow up: attrition <10% vs. ≥10%

### 8.3 Subgroup analysis for functional improvement at longest follow up: follow up ≤6 months vs. >6

### 8.4 Subgroup analysis for functional improvement at longest follow up: indication for rehabilitation

### 8.5 Subgroup analysis for functional improvement at longest follow up: cognitive status in eligibility criteria

Subgroups: (i) included patients with dementia of any severity, (ii) included patients with mild to moderate dementia (excluded severe dementia), (iii) excluded patients with dementia, and (iv) not reported.

## Appendix 9. Functional improvement (as defined by authors) at discharge

## Appendix 10. Discharge home

### 10.1 Subgroup analysis for discharge home: mean age <80 vs. >80

### 10.2 Subgroup analysis for discharge home: attrition <10% vs. >10%

### 10.3 Subgroup analysis for discharge home: indication for rehabilitation

### 10.4 Subgroup analysis for discharge home: geriatrician in team

### 10.5 Subgroup analysis for discharge home: OT in team

### 10.6 Subgroup analysis for discharge home: nurse in team

### 10.7 Subgroup analysis for discharge home: social worker in team

### 10.8 Subgroup analysis for discharge home: cognitive status in eligibility criteria

## Appendix 11. Remaining home at longest follow up

### 11.1 Subgroup analysis remaining home at longest follow up: mean age <80 vs. ≥80

### 11.2 Subgroup analysis remaining home at longest follow up: attrition <10% vs. ≥10%

### 11.3 Subgroup analysis remaining home at longest follow up: follow up <6 vs. >6 months

### 11.4 Subgroup analysis remaining home at longest follow up: indication for rehabilitation

### 11.5 Subgroup analysis remaining home at longest follow up: geriatrician in team

### 11.6 Subgroup analysis remaining home at longest follow up: OT in team

### 11.7 Subgroup analysis remaining home at longest follow up: nurse in team

### 11.8 Subgroup analysis remaining home at longest follow up: social worker in team

### 11.9 Subgroup analysis remaining home at longest follow up: cognitive status in eligibility criteria

Subgroups: (i) included patients with dementia of any severity, (ii) included patients with mild to moderate dementia (excluded severe dementia), (iii) excluded patients with dementia, and (iv) not reported.

## Appendix 12. Length of stay in hospital

### 12.1 Subgroup analysis for length of stay: mean age <80 vs. >80

### 12.2 Subgroup analysis for length of stay: indication for rehabilitation

### 12.3 Subgroup analysis for length of stay: geriatrician in team

### 12.4 Subgroup analysis for length of stay: OT in team

### 12.5 Subgroup analysis for length of stay: nurse in team

### 12.6 Subgroup analysis for length of stay: social worker in team

### 12.7 Subgroup analysis for length of stay: cognitive status in eligibility criteria

Subgroups: (i) included patients with dementia of any severity, (ii) included patients with mild to moderate dementia (excluded severe dementia), (iii) excluded patients with dementia, and (iv) not reported.

## Appendix 13. Cognition at longest follow up

MMSE = mini-mental status examination; SD = standard deviation; MD = mean difference; CI = confidence interval

## Appendix 14. Mood at longest follow up

GDS = Geriatric depression scale.

### 14.1 Subgroup analysis for mood: mean age <80 vs. ≥80

### 14.2 Subgroup analysis for mood: follow up <6 vs. >6 months

### 14.3 Subgroup analysis for mood: indication for rehabilitation

### 14.4 Subgroup analysis for mood: geriatrician in team

### 14.5 Subgroup analysis for mood: OT in team

### 14.6 Subgroup analysis for mood: nurse in team

### 14.7 Subgroup analysis for mood: social worker in team

### 14.8 Subgroup analysis for mood: cognitive status in eligibility criteria

## Appendix 15. Quality of life at longest follow up

GQLQ = geriatric quality of life questionnaire; SF-36 = 36-item short form survey; HRQoL-15D = health-related quality of life 15 dimension instrument; EQ-5D-3L = instrument by EuroQol, 5 dimension, 3 levels; SMD = standardized mean difference.

### 15.1 Subgroup analysis for quality of life: mean age <80 vs. ≥80

### 15.2 Subgroup analysis for quality of life: follow up <6 vs. >6 months

### 15.3 Subgroup analysis for quality of life: measure of quality of life

### 15.4 Subgroup analysis for quality of life: indication for rehabilitation

### 15.5 Subgroup analysis for quality of life: geriatrician in team

### 15.6 Subgroup analysis for quality of life: OT in team

### 15.7 Subgroup analysis for quality of life: nurse in team

### 15.8 Subgroup analysis for quality of life: social worker in team

### 15.9 Subgroup analysis for quality of life: cognitive status in eligibility criteria

## Appendix 16. Supplementary table S1: Comparison of outcome estimates with other systematic reviews of geriatric rehabilitation.

|  | **BMJ 2010** | **Cochrane 2015** | **Cochrane 2021** | **Age Ageing 2023** |
| --- | --- | --- | --- | --- |
| Setting/patient | Inpatient | Day hospital | Inpatient for hip fracture only | Outpatient |
| Comparator | Usual care | Alternative care or no care | Usual care | Usual care, inpatient rehab, home-based rehab, day rehabilitation, primary care, discharge with no support or no follow-up |
| Mortality | Discharge:  RR 0.72 (0.55 to 0.95)  Longest follow up:  RR 0.87 (0.77 to 0.97) | Longest follow up:  OR 1.05 (0.85 to 1.28) | Discharge: RR 0.77  (0.58 to 1.04)  Longest follow up:  RR 0.91 (0.80 to 1.05) | No meta-analysis |
| LTC admission | Discharge:  RR 0.64 (0.51 to 0.81)  Longest follow up:  RR 0.84 (0.72 to 0.99) | Death or LTC admission at longest follow up:  OR 0.85 (0.63-1.14) | Death or LTC admission at longest follow up:  RR 0.88 (0.80 to 0.98)  LTC admission at longest follow up:  RR 0.90 (0.76 to 1.06) |  |
| Functional improvement | Discharge:  OR 1.75 (1.31 to 2.35)  Longest follow up:  OR 1.36 (1.07 to 1.71) |  |  |  |
| Functional status |  | No meta-analysis |  | SMD 0.11 (-0.11 to 0.34), favours intervention |
| Length of stay | No meta-analysis (summarized descriptively).  General geriatric rehab: 24.5 vs 15.1 days (longer in rehab)  Hip fracture rehab: 24.6 vs 28.9 days (shorter in rehab) |  |  | MD -2.41 (-4.31 to -0.22) days, I2 74% (shorter in intervention) |
| Quality of life |  | No meta-analysis | No meta-analysis for overall score | No meta-analysis for overall score |
| Meta-analysis outcomes not reported in our review |  | Death or deterioration in ADL: OR 1.07  (0.76 to 1.49)  Death or poor outcome (institutional care, disability or deterioration): OR 0.92  (0.74 to 1.15)  Deterioration in ADL: OR 1.11  (0.68 to 1.8) | Greater dependence in ADL: RR 0.64 (0.51 to 0.81)  Greater dependence in mobility: RR 0.83 (0.71 to 0.98)  Delirium in hospital: RR 0.85  (0.74 to 0.98). Various other complications reported in fewer than 3 studies.  Readmission to hospital: RR 0.97 (0.84 to 1.12) | Readmission to hospital or LTC: RR 0.90 (0.73–1.10) |

RR = risk ratio, OR = odds ratio, LTC = long-term care, SMD = standardized mean difference, MD = mean difference
